# Metabolite Identification of HIV-1 Capsid Modulators PF74 and 11L in Human Liver Microsomes

**DOI:** 10.3390/metabo12080752

**Published:** 2022-08-16

**Authors:** Shujing Xu, Lin Sun, Dang Ding, Xujie Zhang, Xinyong Liu, Peng Zhan

**Affiliations:** Key Laboratory of Chemical Biology (Ministry of Education), Department of Medicinal Chemistry, School of Pharmaceutical Sciences, Shandong University, 44 West Culture Road, Jinan 250012, China

**Keywords:** PF74, 11L, capsid modulators, human liver microsomes, UPLC–UV–HRMS, metabolite identification

## Abstract

**PF74** and **11L**, as potent modulators of the HIV-1 capsid protein, have been demonstrated to act at both early and late stages in the HIV-1 life cycle. However, their clearance is high in human liver microsomes (HLMs). The main goal of this study was to clarify the metabolism of **PF74** and **11L** in HLMs, and provide guidance for future structural optimization. To accomplish this, the phase-I metabolites of **PF74** and **11L**, resulting from in vitro incubation with HLMs, were investigated via ultra-performance liquid chromatography–ultraviolet–high-resolution mass spectrometry (UPLC–UV–HRMS). The results show that 17 phase-I metabolites were putatively annotated for **PF74**, whereas 16 phase-I metabolites were found for **11L**. The main metabolic pathways of **PF74** in HLMs were oxidation and demethylation, and the secondary metabolic pathway was hydrolysis; thus, the di-oxidation and demethylation products (**M7**, **M9**, **M11**, and **M14**) were found to be major metabolites of **PF74** in HLMs. In comparison, the main metabolic pathways of **11L** in HLMs were oxidation, demethylation, dehydrogenation, and oxidative deamination, with **M6′**, **M11′**, **M15′**, and **M16′** as the main metabolites. We suggest that the indole ring and *N*-methyl group of **PF74**, and the aniline group, benzene ring R1′, *N*-methyl, and methoxy group of **11L**, were the main metabolic soft spots. Therefore, our research illuminates structural optimization options in seeking improved HIV-1 CA modulators.

## 1. Introduction

**Figure 1:**
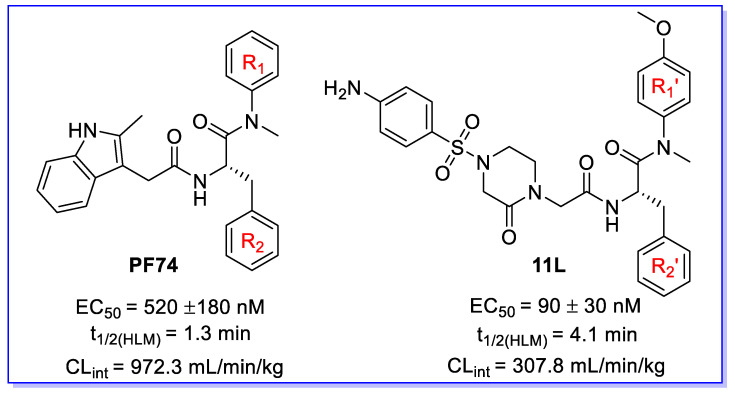
The chemical structures of **PF74** and **11L [10]**. EC_50_ is the concentration of the compound required to achieve 50% protection of TZM-bl cells against HIV-1-induced cytopathic effect, determined in at least triplicate against HIV-1 in TZM-bl cells; t_1/2_ is the half-life; and CL_int_ is the intrinsic clearance.

Human immunodeficiency virus type 1 (HIV-1) remains a worldwide healthcare concern despite the effective development of combination antiretroviral therapy (cART) [1,2]. Existing cART is projected to last a lifetime, and the virus will ultimately choose variants that are resistant to current treatment classes [3,4]. This necessitates the development of innovative medications with diverse resistance profiles and new targets. The multifunctional HIV-1 capsid protein (CA) represents an emerging and highly appealing target in HIV-1 medication research. CA plays essential roles in multiple events during viral replication, such as reverse transcription, nuclear entry, integration site distribution, assembly, and maturation [5,6,7]. Therefore, CA-targeting small molecules could provide antiviral characteristics in both the early and late stages. By far, the most intriguing CA-targeting compound is **PF74**; however, further clinical development of **PF74** is mainly impeded by its inferior metabolic stability [8,9]. Additionally, our previous efforts involved replacing the indole moiety with benzenesulfonamide moiety, and we obtained compound **11L** with increased antiviral activity, but only modestly better microsomal stability [10] (Figure 1). **PF74** and **11L** could affect the morphology and assembly process of viral particles through multiple effects to achieve the purpose of inhibiting viral replication, providing low stoichiometric loading advantages [10,11]. Theoretically, a small molecule could regulate unlimited capsids, which requires CA-targeting modulators with high metabolic stability. Therefore, determining their metabolic soft spots would be essential for directing the optimization process to enhance their pharmacokinetic features.

Metabolite profiling studies of new chemical entities (NCEs) are commonly performed in the pharmaceutical industry to aid in medication development [12,13]. These investigations not only provide information regarding the metabolic fate of NCEs, they also assist in the identification of metabolic soft spots in the parent molecule [14]. Elucidating the metabolic profile may allow for metabolic fortification during drug development efforts, and it is also significant for assessing overall drug safety and efficacy [15,16]. Ultra-performance liquid chromatography–ultraviolet–high-resolution mass spectrometry (UPLC–UV–HRMS) is a vital and effective method for the research of drug metabolism and pharmacokinetics, and it is widely utilized to characterize the structural properties of NCEs and their metabolites [17,18,19,20]. UPLC has a powerful separation capacity, making it one of the most useful tools currently available for separating complicated components [21,22,23]. The relative content of different metabolites can be determined according to the peak area of UV, which is helpful to preliminarily determine the main metabolites [18]. HRMS possesses the qualities of high speed, sensitivity, and selectivity, and it has been extensively employed for online structural analysis and quantitative detection of the components [22,24].

In this study, we investigated the phase-I metabolites and metabolic pathways of **PF74** and **11L** in human liver microsomes (HLMs). **PF74** and **11L** were incubated in the coexistence system of HLMs and NADPH for 60 min at 37 °C. The incubated samples were evaluated by UPLC–UV–HRMS. The relative content of each metabolite was determined according to the UV peak area. The structures of the metabolites were elucidated based on their MS spectra, tandem MS (MS/MS) spectra, fragmentation patterns, and comparison of the primary and secondary mass spectrometry signals with the parent drug. Finally, the metabolic pathways of **PF74** and **11L** were proposed based on metabolite data. In the in vitro study, a positive control was used to monitor the incubation. As described in the Supplementary Materials, 7-ethoxycoumarin (7-EC, 10 μM) was utilized as a positive control for phase-I metabolism to assure that proper incubation conditions were maintained. De-ethylated metabolites were observed in the 7-EC positive control, indicating phase-I metabolic activity in liver microsome incubation system.

## 2. Materials and Methods

### 2.1. Chemicals and Reagents

PF74 (purity > 99%) was obtained from MedChemExpress (Shanghai, China), and the information for 11L (purity > 99%) is provided in Supplementary Materials. In addition, 7-EC (purity > 97%) was purchased from Shanghai Macklin Biochemical Co., Ltd. (Shanghai, China). Acetonitrile, formic acid, water, and ammonium formate were purchased from Fisher Scientific (Fair Lawn, NJ, USA). All of the solvents used in the incubation and chromatographic system were UPLC-grade. Human liver microsomes (batch number: 38295) were obtained from Corning Incorporated (Corning, New York, NY, USA). Nicotinamide adenine dinucleotide phosphate (NADPH), phosphate buffer saline (PBS), and MgCl_2_ were obtained from Shanghai Aladdin Biochemical Technology Co., Ltd. (Shanghai, China).

### 2.2. Sample Preparation

To examine phase-I metabolites in vitro, microsomal incubation was performed with 100 mM phosphate buffer saline (pH 7.4) containing PF74 or 11L (10 μM), HLM (1.0 mg/mL), NADPH (1.0 mM), and MgCl_2_ (3.0 mM), and the reaction volume was 400 μL. Furthermore, 7-EC (10 μM) was utilized as a positive control for phase-I metabolism to guarantee that proper incubation conditions were maintained. Incubation without samples was used as a blank control. After preincubation for 5 min at 37 °C, the reactions were started by adding NADPH. After incubation for 60 min at 37 °C, the reactions were quenched by adding 800 μL of acetonitrile solution containing 0.1% formic acid. Afterward, the supernatants were obtained under centrifugation at 13,000 rpm for 10 min, and were then dried under nitrogen gas and the residues redissolved in 200 μL water/acetonitrile solution (*v*:*v* = 10:3). Aliquots were taken at 0 and 60 min, then, the sample (10 μL) was injected into the UPLC–UV–HRMS system for metabolite profiling and identification.

### 2.3. Chromatographic and Mass Spectrometric Methods

#### 2.3.1. Chromatographic and Mass Spectrometric Methods of PF74

The UPLC–UV–HRMS analysis was carried out using an ACQUITY UPLC system hyphenated to a Thermo Q-Exactive HF tandem mass spectrometer equipped with an electrospray ionization interface that operated in positive ion mode.

Chromatographic Method: The chromatography was performed on an ACQUITY UPLC HSS T3 column (2.1 × 100 mm, 1.8 µm; Waters Corporation, MA, USA) maintained at 40 °C. Automatic injector temperature maintained at 8 °C. The mobile phase, made up of water/acetonitrile (*v*:*v* = 95:5) solution containing 0.1% formic acid and 2 mM ammonium formate (A), and water/acetonitrile (*v*:*v* = 5:95) solution containing 0.1% formic acid and 2 mM ammonium formate (B), was delivered at a flow rate of 300 µL/min. The gradient procedures were optimized as follows: 2% B at 0–2 min, 2–45% B at 2–6 min, 45–75% B at 6–12 min, 75–90% B at 12–16 min, 90% B at 16–18 min, and finally, 2% B at 18–20 min. The injection volume was 10 μL. The UV detection was performed using a photodiode array detector, and wavelength scanned from 190 to ~500 nm.

Mass Spectrometric Method: The electrospray ionization source parameters were set as follows: capillary temperature, 375 °C; spray voltage, 3.5 kV; sheath gas flow rate, 40 L/h; auxiliary gas flow rate, 15 L/h; auxiliary gas heater temperature, 300 °C; S-Lens voltage, 55 V. The data were collected in the *m*/*z* range of 140–1400 Da (full mass scan) and 200–2000 Da (MS/MS scan) in centroid mode. The collision energy was 10/15 V. The mass resolutions for full mass scans and MS/MS scans were chosen to be above 60,000 and 15,000/30,000, respectively. Collision-induced dissociation (CID) was utilized to attain the MS/MS fragment of PF74 and its metabolites.

#### 2.3.2. Chromatographic and Mass Spectrometric Methods of 11L

The UPLC–UV–HRMS analysis was carried out using an ACQUITY UPLC system hyphenated to a Thermo Q-Exactive HF tandem mass spectrometer equipped with an electrospray ionization interface that operated in positive or negative ion mode.

Chromatographic Method: The chromatography was performed on an ACQUITY UPLC HSS T3 column (2.1 × 100 mm, 1.8 µm; Waters Corporation, MA, USA) maintained at 40 °C. The automatic injector temperature was maintained at 8 °C. The mobile phase, made up of water/acetonitrile (*v*:*v* = 95:5) solution containing 0.1% formic acid and 2 mM ammonium formate (A), and water/acetonitrile (*v*:*v* = 5:95) solution containing 0.1% formic acid and 2 mM ammonium formate (B), was delivered at a flow rate of 500 µL/min. The gradient procedures were optimized as follows: 2% B at 0–2 min, 2–55% B at 2–12 min, 55–90% B at 12–16 min, 90% B at 16–18 min, and finally, 2% B at 18–20 min. The injection volume was 10 μL. UV detection was performed using a photodiode array detector, and wavelength scanned from 190 to ~500 nm.

Mass Spectrometric Method: The electrospray ionization source parameters were set as follows: capillary temperature, 375 °C; spray voltage, 3.5 kV (+), 3.0 kV (−); sheath gas flow rate, 40 L/h; auxiliary gas flow rate, 15 L/h; auxiliary gas heater temperature, 300 °C; S-Lens voltage, 55 V. The data were collected in the *m*/*z* range of 140–1400 Da (+, full mass scan), 120–1400 (−, full mass scan), and 200–2000 Da (MS/MS scan) in centroid mode. The collision energy was 10/15/20 V (+), 10/20/30 V (−). The mass resolutions for full mass scans and MS/MS scans were chosen to be above 60,000 and 15,000, respectively. Collision-induced dissociation (CID) was utilized to attain the MS/MS fragment of 11L and its metabolites.

### 2.4. Data Processing

The acquired data sets were captured and analyzed by Thermo Xcaliber 4.2 workstation (Thermo Scientific, Bremen, Germany). The possible element composition was speculated by accurate molecular mass, and the data were processed by mass spectrometry fragmentation information. The molecular formulae of parent ions and fragment ions were predicted using a molecular formula prediction module, and the structures of metabolites, in vitro, of PF74 and 11L were identified. The maximum mass errors between the measured and the theoretical value were limited to 5 ppm.

## 3. Results and Discussion

### 3.1. Identification of PF74 Metabolites and Proposed Metabolic Pathways in HLMs

**Figure 2:**
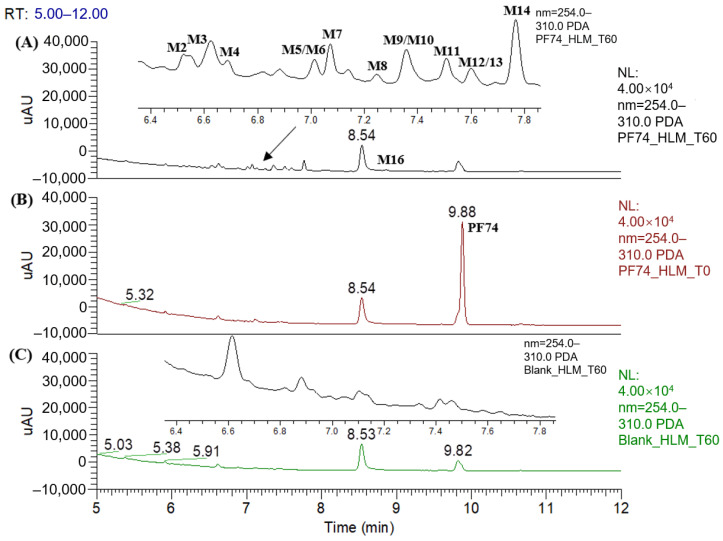
UPLC–UV (254–310 nm) chromategrams of **PF74** in HLMs: (**A**) UPLC–UV spectrum of T60 (M1 and M15 are not listed due to weak UV absorption); (**B**) UPLC–UV spectrum of T0; (**C**) blank UPLC–UV spectrum.

**Figure 3:**
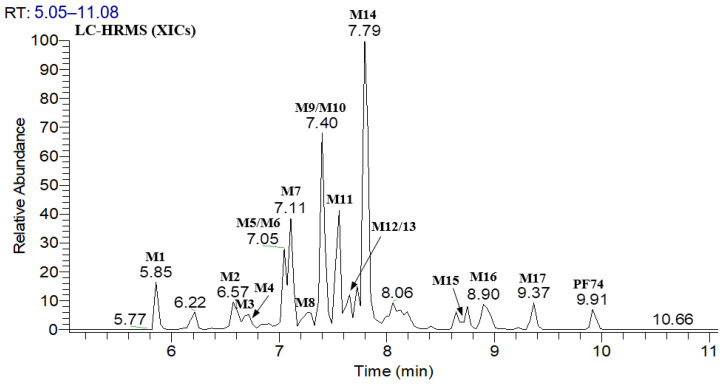
XIC of **PF74** in HLMs.

Figure 2 displays the UPLC–UV chromatogram of **PF74** in HLMs at the detection wavelength of 254–310 nm, and Figure 3 displays the extracted ion chromatogram (XIC) of **PF74** in HLMs. Metabolites were tentatively identified by comparing the UPLC–UV and UPLC–HRMS spectra and retention times of potential metabolites to the parent compound **PF74**. It is worth noting that after incubation at 37 °C for 60 min, **PF74** was almost completely metabolized in HLMs. A total of 17 phase-I metabolites were discovered in the HLMs, and their spectra were compared to **PF74** for structural confirmation (Figure 2 and Figure 3). According to the UV peak area, **M7**, **M9**, **M11**, and **M14** were preliminarily identified as the main metabolites (Figure 2 and Table 1).

**Figure 4:**
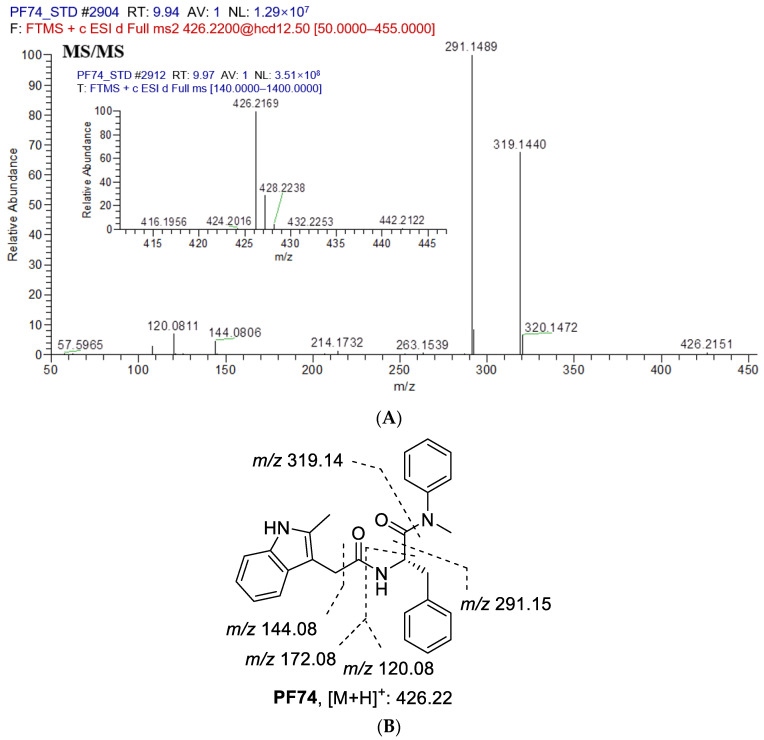
(**A**) Collision induced dissociation (CID) mass spectrometry of **PF74**; (**B**) Analysis of mass spectrometry fragments (*m*/*z* 426.2151). Here are major fragment ions (*m*/*z* 319.1440, 291.1489, 172.0762, 144.0806, 120.0811) of **PF74** without metabolism.

The first step in identifying the structures of metabolites was to examine the MS/MS fragmentation pattern of **PF74**. The MS/MS fragmentation pattern of **PF74** was studied in positive mode. A protonated molecular ion [M + H]^+^ of **PF74** at *m*/*z* 426.2151 was found in the full-scan mass spectrum. The protonated molecules then produced a series of characteristic fragment ions at *m*/*z* 320.1472, 319.1440, 291.1489, 263.1539, 214.1732, 172.0762, 144.0806, 120.0811 and 57.5965. **PF74** was eluted at 9.91 min under the experimental conditions. The MS/MS product ion spectrum and predominant fragmentation patterns of **PF74** were depicted in Figure 4.

#### 3.1.1. Metabolite Profiling of PF74 in HLMs

A list of all the putative metabolites of **PF74** with the proposed type of biotransformation and elemental composition, as well as the retention time, relative abundance, UV peak area, accurate mass of the protonated molecule and mass error, were summarized in Table 1 and Table 2. UPLC–MS/MS analysis of unchanged **PF74** and its 17 metabolites yielded informative and prominent product ions for structural characterization (Figure S2).

##### Amide Hydrolysis Metabolite (M1)

**M1**, eluted at 5.85 min, gave rise to its [M + H]^+^ ion at *m*/*z* 255.1490 (C_18_N_6_H_12_O). It was 171 u lower than that of **PF74**, and produced secondary fragment ions at *m*/*z* 120.08, indicating that it might be the amide bond adjacent to the indole ring hydrolyzed to the amino (−171 u) product (Figure S2A).

##### Tri-oxidation and Demethylation Metabolites (M2, M3, M6, M8, M10, and M12)

Metabolites **M2**, **M3**, **M6**, **M8**, **M10**, and **M12** exhibited retention times at 6.57, 6.61, 7.05, 7.29, 7.40, and 7.65 min, respectively, and they displayed the same theoretical protonated ion [M + H]^+^ at *m*/*z* 460.1867 (C_26_H_25_N_3_O_5_). This is 34u higher than **PF74**, indicating that they might be tri-oxidation (+48 u) and *N*-demethylation (−14 u) products. Metabolites **M2**, **M3**, **M6**, **M8**, and **M10** lost one molecule of water to form secondary fragment ions at *m*/*z* 442.18. Metabolites **M2**, **M3**, and **M10** had secondary fragment ions at *m*/*z* 204.07, 186.05, and 162.05, suggesting that the indole ring was oxidized. Among them, **M2** and **M10** had secondary fragment ions at *m*/*z* 351.13, 323.14, 190.09, and 120.08, indicating that benzene ring R_2_ did not undergo oxidation. In accordance with the secondary fragment ion at *m*/*z* 257.13, it is speculated that benzene ring R_1_ underwent mono-oxidation and *N*-demethylation (Figure S2B,J). Additionally, the secondary fragment ions at *m*/*z* 206.08 and 136.08 of **M3** suggest that the mono-oxidation of benzene ring R_2_ had occurred (Figure S2C). Metabolite **M6** had secondary fragment ions at *m*/*z* 188.07 and 170.06, indicating that the indole ring was mono-oxidized, and the secondary fragment ions at *m*/*z* 367.13, 339.13, 222.08, and 152.07 indicate that the benzene ring R_2_ was di-oxidized (Figure S2F). Secondary fragment ions at *m*/*z* 202.05 and 186.05 suggest that the indole ring of metabolite **M8** underwent oxidation (Figure S2H). In accordance with the secondary fragment ions at *m*/*z* 367.13 and 339.13 of **M12**, the oxidation of the indole ring and benzene ring R_2_ is also speculated (Figure S2L).

##### Tri-oxidation Metabolites (M4 and M5)

Metabolites **M4** and **M5** possessed the same theoretical protonated molecular ion [M + H]^+^ at *m*/*z* 474.2024 (C_27_H_27_N_3_O_5_), and were detected at 6.68 and 7.05 min, respectively. They were 48 u higher than that of **PF74**; therefore, they could be the tri-oxidation products. The secondary fragment ion at *m*/*z* 456.19 was formed when they lost one molecule of water. The metabolite **M4** had secondary fragment ions at *m*/*z* 188.07 and 170.06, demonstrating that the indole ring was mono-oxidized. The secondary fragment ions at *m*/*z* 222.08 and 152.07 show that the benzene ring R_2_ was di-oxidized, and *m*/*z* 108.08 show that the benzene ring R_1_ was not oxidized (Figure S2D). The secondary fragment ions at *m*/*z* 202.05 and 174.06 in the metabolite **M5** indicate that the indole ring was tri-oxidized, and the secondary fragment ions at *m*/*z* 120.08 and 108.08 indicate that the benzene rings R_1_ and R_2_ were not oxidized (Figure S2E).

##### Di-oxidation and Demethylation Metabolites (M7, M11, M13, M14, and M16)

Metabolites **M7**, **M11**, **M13**, **M14**, and **M16**, with their retention times of 7.11, 7.56, 7.65, 7.79, and 8.90 min, respectively, were 18 u higher than that of **PF74**. They displayed the same theoretical protonated molecular ion [M + H]^+^ at *m*/*z* 444.1918 (C_26_H_25_N_3_O_4_), which indicates that they were di-oxidation (+32 u) and demethylation (−14 u) products. The metabolites **M7** and **M13** had secondary fragment ions at *m*/*z* 188.07 and 170.06, Implying that the indole ring was mono-oxidized. Secondary fragment ions at *m*/*z* 120.08 indicate that benzene ring R_2_ was not oxidized, while secondary fragment ions at *m*/*z* 257.13 suggest that benzene ring R_1_ was mono-oxidized and *N*-demethylated (Figure S2G,M). The metabolites **M11**, **M14**, and **M16** had secondary fragment ions at *m*/*z* 204.07, 186.06, and 162.06, and we speculate that the indole ring was di-oxidized (Figure S2K,N,P).

##### Di-oxidation Metabolite (M9)

Metabolite **M9**, eluted at 7.40 min, possessed the experimental [M + H]^+^ ion at *m*/*z* 458.2074 (C_27_H_27_N_3_O_4_), which was 32 u higher than that of **PF74**, meaning that it might be a di-oxidation (+32 u) product of **PF74**. Secondary fragment ions at *m*/*z* 204.07 and 162.05 indicate that the indole ring was oxidized, and *m*/*z* 190.09 and 108.08 indicate that the benzene rings R_1_ and R_2_ were not oxidized. Additionally, *m*/*z* 440.20, 333.12, and 305.13 were a series of fragments formed by oxidative dehydration (Figure S2I).

##### Mono-oxidation Metabolite (M15)

Metabolite **M15** with a protonated ion [M + H]^+^ at *m*/*z* 442.2134 (C_27_H_27_N_3_O_3_), was eluted at 8.75 min. It was 16 u higher than that of **PF74**, showing that there was a mono-oxidation product. The secondary fragment ion at *m*/*z* 188.07 determines that the indole ring was mono-oxidized, and *m*/*z* 424.20, 317.13, 289.13, and 170.06 were a series of fragments formed by oxidative dehydration (Figure S2O).

##### Mono-oxidation and Demethylation Metabolite (M17)

Metabolite **M17**, 2 u higher than that of **PF74**, possessed a protonated molecule ion [M + H]^+^ at *m*/*z* 428.1969 (C_26_H_25_N_3_O_3_) with the retention time of 9.37 min, suggesting that **M17** was the product of mono-oxidation (+16 u) and demethylation (−14 u). In addition, *m*/*z* 188.07 leads us to speculate that the indole ring was mono-oxidized, and *m*/*z* 410.19, 317.18, 289.13, and 170.06 were a series of fragments formed by oxidative dehydration (Figure S2Q).

#### 3.1.2. Proposed Metabolic Pathways of PF74

**Figure 5:**
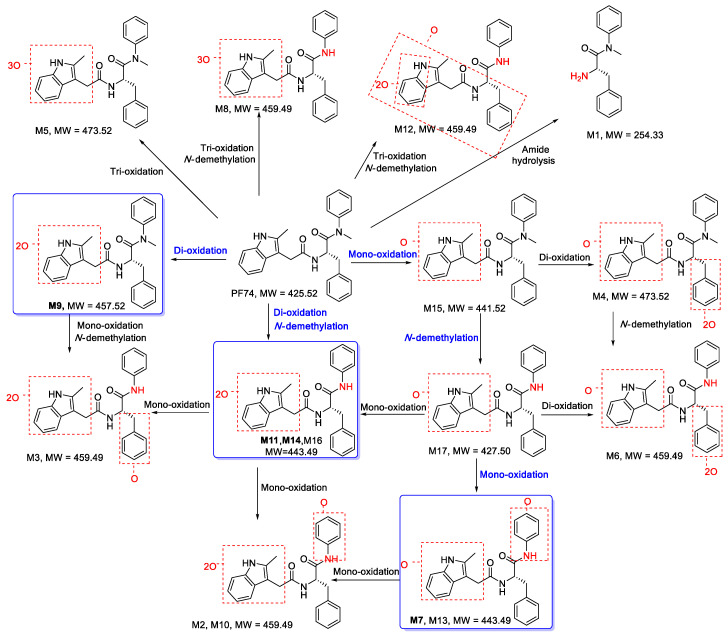
Proposed metabolic pathways of **PF74** in HLMs. The main pathways are highlighted in blue.

In this investigation, a total of 17 metabolites were found in HLMs of **PF74**. The proposed metabolic pathways of **PF74** are exhibited in Figure 5. There was a series of metabolic reactions containing mono-oxidation, di-oxidation, tri-oxidation, mono-oxidation and demethylation, di-oxidation and demethylation, tri-oxidation and demethylation, amide hydrolysis, and so on. Metabolites **M7**, **M11**, **M14** (di-oxidation and *N*-demethylation products), and **M9** (di-oxidation product) were the four most abundant metabolites, so the main metabolic pathways of **PF74** in HLMs were di-oxidation and *N*-demethylation, which also suggests that the indole ring, benzene ring R_1_, and *N*-methyl group were the main metabolic soft spots. In a previous study by Wang et al. [25], upon substituting the electron-rich indole ring of **PF74** with a less electron-rich benzamide group, a preferred compound with similar submicromolar potency and much longer (51-fold) half-life was reported. Additionally, by replacing the benzene ring R_1_ with the electron-deficient benzothiazole moiety, we found a compound that exhibited markedly improved metabolic stability in HLMs, with a half-life 109-fold that of **PF74** [26]. Furthermore, Sahani et al. [27] reported a PF74-like analog with 39 times enhanced metabolic stability over **PF74**, which led to increased resistance to *N*-methylation conceived via building the phenylalanine carboxamide into a pyridine ring. These results are consistent with our current findings, wherein we have identified the phase-I oxidative and *N*-demethylated metabolites as major metabolites of **PF74** in HLMs.

### 3.2. Identification of 11L Metabolites and Proposed Metabolic Pathways in HLMs

**Figure 6:**
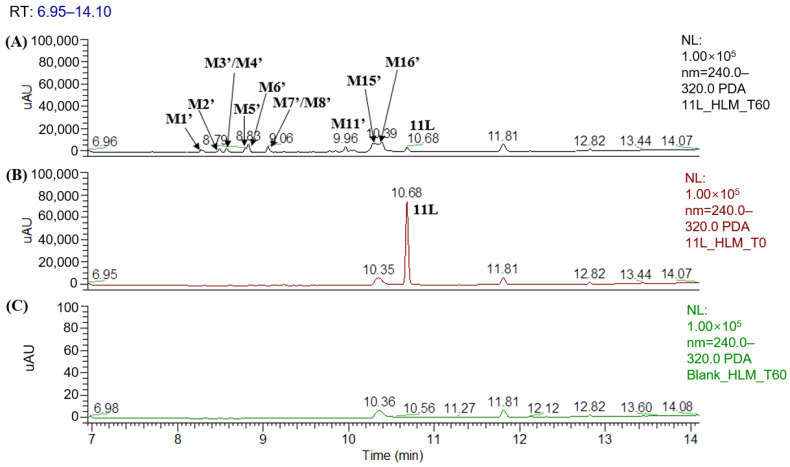
UPLC–UV (254–310 nm) chromatograms of **11L** in HLMs: (**A**) UPLC–UV spectrum of T60; (**B**) UPLC–UV spectrum of T0; (**C**) blank UPLC–UV spectrum.

**Figure 7:**
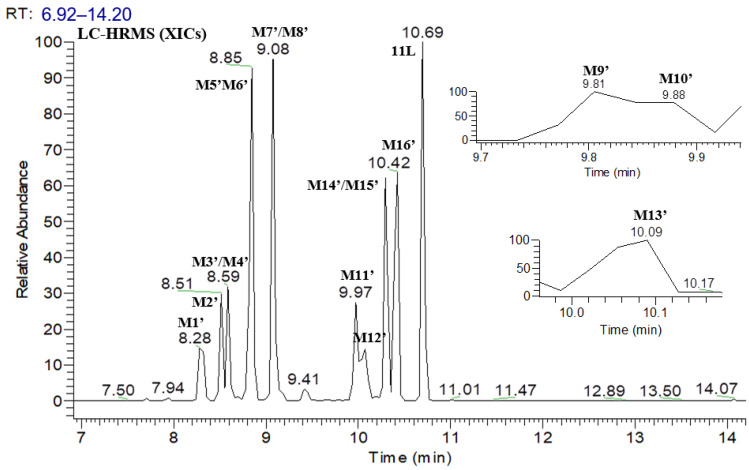
XIC of **11L** in HLMs.

**Figure 8:**
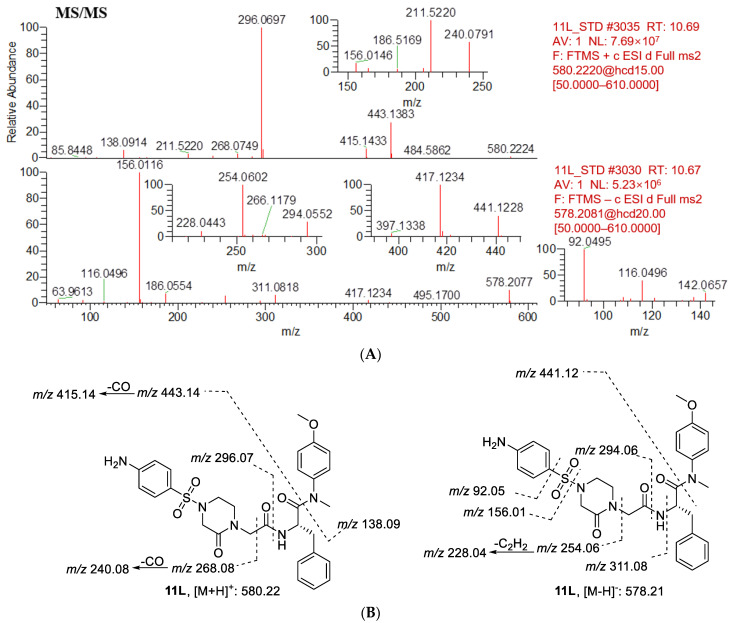
(**A**) CID mass spectrometry of **11L**; (**B**) analysis of mass spectrometry fragments (positive ion, *m*/*z* 580.2224; negative ion, *m*/*z* 578.2077). The major fragment ions of **11L** without metabolism are positive ions at *m*/*z* 443.1384, 296.0697, 268.0749, 138.0914, and negative ions at *m*/*z* 441.1228, 311.0818, 294.0552, 254.0602, 156.0116, 92.0495.

Figure 6 shows the UPLC–UV chromatogram of **11L** in HLMs at the detection wavelength of 240–320 nm, and Figure 7 show the XIC of **11L** in HLMs. Metabolites were tentatively identified by comparing the UPLC–UV and UPLC–HRMS spectra and retention times of potential metabolites to the parent compound **11L**. After incubation at 37 °C for 60 min, the relative abundance of **11L** in HLMs was 7.11%, which explains why **11L** (t_1/2_ = 4.1 min) had a slightly longer half-life than **PF74** (t_1/2_ = 1.3 min). A total of 16 metabolites were discovered in HLMs, and their spectra were compared with those of **11L** for structural confirmation (Figure 7 and Figure 8). According to the UV peak area, **M6′**, **M11′**, **M15′**, and **M16′** are preliminarily identified as the main metabolites (Figure 6 and Table 3). 

The first step in identifying the structures of metabolites was to examine the MS/MS fragmentation pattern of parent compound **11L**. The MS/MS fragmentation pattern of **11L** was examined in positive and negative mode. A protonated molecular ion [M + H]^+^ of **11L** at *m*/*z* 580.2224 and a deprotonated molecular ion [M − H]^−^ of **11L** at *m*/*z* 578.2077 were observed in the full-scan mass spectrum. The protonated molecules then produced a series of characteristic fragment ions at *m*/*z* 443.1383, 415.1433, 296.0697, 268.0749, 240.0791, 211.5220, and 138.0914, and the deprotonated molecules then produced a series of characteristic fragment ions at *m*/*z* 441.1228, 417.1234, 311.0818, 294.0552, 254.0602, 228.0443, 186.0554, 156.0116, and 92.0495. Moreover, **11L** was eluted at 10.69 min under the experimental conditions. The MS/MS product ion spectrum and predominant fragmentation patterns of **11L** are displayed in Figure 8.

#### 3.2.1. Metabolite Profiling of 11L in HLMs

A list of all the putative metabolites of **11L** with the proposed type of biotransformation and elemental composition, as well as the retention time, relative abundance, UV peak area, accurate mass of the protonated (or deprotonated) molecule, and mass error, are summarized in Table 3 and Table 4. UPLC–MS/MS analysis of unchanged **11L** and its 16 metabolites yielded informative and prominent product ions for structural characterization (Figure S3).

##### Tri-oxidation Metabolite (M1′)

Metabolite **M1′**, eluted at 8.29 min, possessed the experimental protonated molecular ion [M + H]^+^ at *m*/*z* 628.2078 (C_29_H_33_N_5_O_9_S), which was 48 u higher than that of **11L**, meaning that it might be a tri-oxidation (+48 u) product of **11L**. The secondary fragment ion at *m*/*z* 284.07 suggests that the benzene ring R_1′_ had not been oxidized, whereas *m*/*z* 138.09 is evidence of the di-oxidation of benzene ring R_2′_ (Figure S3A).

##### Di-oxidation Metabolites (M2′, M6′, M13′)

Metabolites **M2′** and **M6′** were observed at retention times of 8.49 and 8.82 min, respectively, with the same experimental protonated molecular ion [M + H]^+^ at *m*/*z* 612.2123 (C_29_H_33_N_5_O_8_S). The two metabolites were 32 u higher than **11L**, suggesting they were the di-oxidation products. The secondary fragment ions at *m*/*z* 268.08 and 138.09 of the metabolite **M2′** indicate that benzenesulfonamide piperazinone and benzene ring R_1′_ did not undergo oxidation, and *m*/*z* 475.13 and 296.07 indicate that benzene ring R_2′_ underwent di-oxidation (Figure S3B). The secondary fragment ion at *m*/*z* 172.01 of metabolite **M6′** demonstrates that aniline was mono-oxidized, and *m*/*z* 475.13 and 312.07 indicate that benzene ring R_2′_ was mono-oxidized (Figure S3F).

Furthermore, **M13′** was detected at a retention time of 10.06 min with the experimental deprotonated ion [M − H]^−^ at *m*/*z* 610.1976 (C_29_H_33_N_5_O_8_S), which was 32 u higher than that of **11L**, meaning that it might be a di-oxidation (+32 u) product of **11L**. The secondary fragment ion at *m*/*z* 172.01 of metabolite **M13′** shows that aniline was mono-oxidized, and *m*/*z* 270.06 and 172.01 show that piperazinone was mono-oxidized (Figure S3M).

##### Demethylation and Mono-oxidation Metabolites (M3′, M9′, M10′, M11′)

Metabolites **M3′** and **M11′** produced the same theoretical protonated ions [M + H]^+^ at *m*/*z* 582.2017 (C_28_H_31_N_5_O_7_S), which were eluted at 8.56 min and 9.98 min, respectively. They were 2 u higher than **11L**, and we speculate that metabolites **M3′** and **M11′** were demethylation (−14 u) and mono-oxidation (+16 u) products. The secondary fragment ion at *m*/*z* 140.07 of **M3′** indicates that benzene ring R_1′_ was mono-oxidized and demethylated, and *m*/*z* 268.08, 296.07, and 443.14 indicate that benzene sulfonamide piperazinone and benzene ring R_2′_ were not oxidized (Figure S3C). The secondary fragment ion at *m*/*z* 172.01 of **M11′** implies the mono-oxidation of aniline, and *m*/*z* 122.06 indicates oxidative demethylation (Figure S3K).

Moreover, **M9′** and **M10′**, with their retention times of 9.78 and 9.83 min, respectively, were 2 u higher than that of **11L**. They displayed the same theoretical deprotonated ion [M − H]^−^ at *m*/*z* 580.1871 (C_28_H_31_N_5_O_7_S), which indicates that they were demethylation (−14 u) and mono-oxidation (+16 u) products. The secondary fragment ion at *m*/*z* 156.01 suggests that aniline was not oxidized (Figure S3I,J).

##### Demethylation and Di-oxidation Metabolite (M4′)

Metabolite **M4′**, 18u higher than that of **11L**, presented an experimental protonated ion [M + H]^+^ at *m*/*z* 598.1962 (C_28_H_31_N_5_O_8_S) with the retention time of 8.60 min, which indicates that it was the product of demethylation (−14 u) and di-oxidation (+32 u). The secondary fragment ion at *m*/*z* 172.01 shows that aniline was mono-oxidized, whereas *m*/*z* 475.13 and 312.07 show that the benzene ring R_2′_ was mono-oxidized, and *m*/z 122.06 suggests oxygen demethylation (Figure S3D).

##### *N*-demethylation and *O*-demethylation Metabolite (M5′)

Metabolite **M5′** was eluted at 8.79 min with an experimental protonated ion [M + H]^+^ at *m*/*z* 552.1917 (C_27_H_29_N_5_O_6_S). It was 28u lower than that of **11L**, showing that was a product of *N*-demethylation (−14 u) and *O*-demethylation (−14 u). The secondary fragment ions at *m*/*z* 443.14, 296.07 and 268.08 indicate that only the demethylation reaction occurred in **M5′** (Figure S3E).

##### Mono-oxidation Metabolites (M7′, M16′)

Metabolites **M7′** and **M16′** were detected at 9.05 and 10.36 min, and possessed the same theoretical protonated ions [M + H]^+^ at *m*/*z* 596.2174 (C_29_H_33_N_5_O_7_S), which were 16 u higher than that of **11L**, and we speculate that metabolites **M7′** and **M16′** were mono-oxidized (+16 u) products. The secondary fragment ions at *m*/*z* 268.08 and 138.09 of **M7′** indicate that benzenesulfonamide piperazinone and benzene ring R_1′_ were not oxidized, and *m*/*z* 459.13 and 296.07 indicate that benzene ring R_2′_ was mono-oxidized (Figure S3G). The *m*/*z* 312.07 and 284.07 of **M16′** indicate the mono-oxidation of benzenesulfonamide piperazinone (Figure S3P).

##### *N*-demethylation Metabolite (M8′)

Metabolite **M8′**, possessing an experimental protonated ion [M + H]^+^ at *m*/*z* 566.2041 (C_28_H_31_N_5_O_6_S), was eluted at 9.08 min, which was 14 u higher than **11L**. Due to the secondary fragment ions at *m*/*z* 124.08, **M8′** is presumed to be the product of *N*-demethylation (−14 u) (Figure S3H).

##### Dehydrogenation and Mono-oxidation Metabolite (M12′)

Metabolite **M12′**, eluted at 10.04 min, possessed the experimental protonated ion [M + H]^+^ at *m*/*z* 594.2021 (C_29_H_31_N_5_O_7_S), which was 14 u higher than that of **11L**, meaning that it might be a dehydrogenated (−2 u) and mono-oxidized (+16 u) product. The secondary fragment ion at *m*/*z* 172.01 of **M12′** indicates the mono-oxidation of aniline, and *m*/*z* 254.06 and 172.01 indicate the reductive dehydrogenation of piperazinone (Figure S3L).

##### Demethylation Metabolite (M14′)

Metabolite **M14′**, 14 u lower than that of **11L**, presented an experimental protonated ion [M + H]^+^ at *m*/*z* 566.2068 (C_28_H_31_N_5_O_6_S) with the retention time of 10.29 min, suggesting that **M14′** was the product of demethylation (−14 u). The secondary fragment ions at *m*/*z* 443.14, 296.07, 268.08, and 156.01 indicate that only the demethylation reaction occurred in **M14′** (Figure S3N).

##### Oxidative Deamination and Mono-oxidation Metabolite (M15′)

Metabolite **M15′** was eluted at 10.32 min with an experimental protonated ion [M + H]^+^ at *m*/*z* 597.2002 (C_29_H_32_N_4_O_8_S). It was 17u higher than that of **11L**, showing that it was a product of oxidative deamination (+16 u and −15 u) and mono-oxidation (+16 u). The *m*/*z* 313.05 and 285.05 indicate that mono-oxidation and oxidative deamination of benzenesulfonamide piperazinone. The *m*/*z* 579.21 was the secondary fragment ion obtained by oxidative dehydration (Figure S3O).

#### 3.2.2. Proposed Metabolic Pathways of 11L

**Figure 9:**
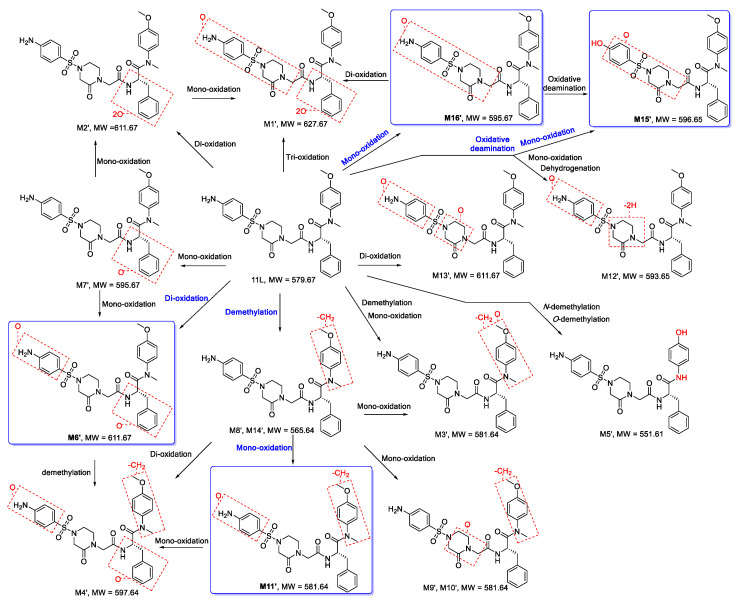
Proposed metabolic pathways of **11L** in HLMs. The main pathways are highlighted in blue.

On the basis of the MS/MS spectra and fragmentation patterns, the metabolic pathways of **11L** are accordingly proposed, as shown in Figure 9. The metabolic reactions are summarized as follows: mono-oxidation, di-oxidation, tri-oxidation, demethylation, mono-oxidation and demethylation, mono-oxidation and oxidative deamination, mono-oxidation and dehydrogenation, *N*- demethylation and *O*-demethylation. Metabolite **M6′** (di-oxidation product), **M11′** (mono-oxidation and demethylation product), **M15′** (mono-oxidation and oxidative deamination product), and **M16′** (mono-oxidation product) were the four metabolites with the highest relative abundance. Therefore, oxidation, demethylation, and oxidative deamination were the main metabolic pathways of **11L** in HLMs, which also indicates that the aniline group, benzene ring R1′, *N*-methyl, and methoxy group of **11L** were the main metabolic soft spots. It has been reported that the introduction of chlorine and fluorine atoms can block metabolically labile sites and improve the metabolic stability of compounds [28]. Recently, Sahani et al. [29] found that the substitution of a methoxy group on benzene ring R1′ and an amino group on aniline via chlorine atoms could slightly improve the metabolic stability of 11L-like analogs. Notably, lenacapavir, a long-acting capsid modulator currently in clinical phase III, contains a core structure similar to **PF74** and **11L** [30]. However, its left wing introduced a fluorine-rich tetrahydrocyclopenta-pyrazole ring, and replaced the benzene ring R1 (R1′) with an indazole ring, as well as introducing the pyridine group to avoid *N*-methylation [31]. The incorporation of electron-withdrawing groups (halogens and sulfonyls), metabolically stable ring systems (cyclopentane and pyrazoles), and rigidifying elements might be the reasons for its high metabolic stability.

## 4. Conclusions

The primary goal of metabolite screening in early drug development is to detect metabolic soft spots that result in poor metabolic stability. This information can direct synthetic chemistry efforts through the identification of compounds with desirable pharmacokinetic characteristics. In this study, phase-I metabolites, and possible metabolic pathways of **PF74** and **11L** in HLMs, were identified for the first time. According to the ionization and fragmentation rules of ESI-MS, the metabolites were identified by UPLC–UV–HRMS data based on the characteristic fragments and characteristic neutral loss of **PF74** and **11L**. For **PF74**, the oxidation of the indole ring and *N*-demethylation of the parent compound were the main transformations. For **11L**, oxidation of the aniline group and benzene ring R1′, demethylation, dehydrogenation, and oxidative deamination metabolites were the relatively most intense. Therefore, it may be an effective method to improve the metabolic stability of compounds by substituting electron-deficient groups for electron-rich indole ring and aniline groups, and substituting benzo-aromatic heterocyclic rings for benzo-methoxy groups to overcome the defects of oxidation and demethylation. In summary, this study’s findings of metabolic transformation information will open up prospects for structural optimization, leading to the identification of novel, more metabolically stable HIV-1 CA modulators.

## Figures and Tables

**Table 1:** List of putative metabolites of **PF74** with the primary identification parameters and hypothesized biotransformation. The relatively most intense metabolites are in bold.

Metabolite	[M + H]^+^ *m*/*z*	Retention Time (min)	Relative Abundance (UV Peak Area%)	UV Peak Area (254–310 nm)	Type of Biotransformation	Diagnostic Ions
M1	255.1490	5.85	+	+	Amide hydrolysis (P − C_11_H_9_NO)	255.15, 120.08
M2	460.1866	6.57	4.26	1269	Tri-oxidation and demethylation (P + 3O − CH_2_)	460.19, 442.18, 351.13, 323.14, 257.13, 204.07, 190.09, 186.05, 162.05, 120.08
M3	460.1866	6.61	4.71	1401	Tri-oxidation and demethylation (P + 3O − CH_2_)	460.19, 442.18, 206.08, 204.07, 186.05, 162.05, 136.08
M4 #	474.2017	6.68	3.54	1055	Tri-oxidation (P + 3O)	474.20, 456.19, 222.08, 188.07, 170.06, 152.07, 108.08
M5 *	474.2028	7.05	4.97	1480	Tri-oxidation (P + 3O)	474.20, 456.19, 202.05, 174.06, 120.08, 108.08
M6 *	460.1866	7.05	2.39	713	Tri-oxidation and demethylation (P + 3O − CH_2_)	460.19, 442.18, 367.13, 339.13, 222.08, 188.07, 170.06, 152.07
**M7**	**444.1917**	**7.11**	**13.73**	**4088**	**Di-oxidation and demethylation (P + 2O − CH_2_)**	**444.19, 257.13, 188.07, 170.06, 120.08**
M8	460.1867	7.29	2.67	796	Tri-oxidation and demethylation (P + 3O − CH_2_)	460.19, 442.18, 202.05, 186.05
M9 *	**458.2074**	**7.40**	**13.48**	**4013**	**Di-oxidation** **(P + 2O)**	**458.21, 440.20, 333.12, 305.13, 204.07, 190.09, 162.05, 108.08**
M10 *	460.1863	7.40	2.87	854	Tri-oxidation and demethylation (P + 3O − CH_2_)	460.19, 442.20, 351.13, 323.14, 257.13, 204.07, 190.09, 186.05, 162.05, 120.08
**M11**	**444.1917**	**7.56**	**9.81**	**2919**	**Di-oxidation and demethylation (P + 2O − CH_2_)**	**444.19, 204.07, 186.06, 162.05**
M12 *	460.1864	7.65	1.35	403	Tri-oxidation and demethylation (P + 3O − CH_2_)	460.19, 367.13, 339.13
M13 *	444.1918	7.65	4.94	1471	Di-oxidation and demethylation (P + 2O − CH_2_)	444.19, 257.13, 188.07, 170.06, 120.08
**M14**	**444.1915**	**7.79**	**25.62**	**7627**	**Di-oxidation and demethylation (P + 2O − CH_2_)**	**444.19, 204.07, 186.06, 162.05**
M15	442.2134	8.75	+	+	Mono-oxidation (P + O)	442.21, 424.20, 317.13, 289.13, 170.06
M16	444.1919	8.90	4.29	1279	Di-oxidation and demethylation (P + 2O − CH_2_)	444.19, 204.07, 186.06, 162.06
M17	428.1969	9.37	1.34	399	Mono-oxidation and demethylation (P + O − CH_2_)	428.20, 410.19, 317.18, 289.13, 188.07, 170.06
PF74 (Parent)	426.2169	9.91	+	+	NA	426.22, 319.14, 291.15, 172.08, 144.08, 120.08

The semiquantitative data of UV (254–310 nm) peak area are shown in the table: P, parent; ND, not detected; +, the signal was detected in UPLC–HRMS, but the UV signal was too weak to integrate the peak area at 254–310 nm; #, peak area is the value after deducting blank matrix; *, the metabolite peaks overlapped, and the UV peak area was calculated and allocated according to the ratio of the mass spectrum peak area; NA, not applicable. Relative abundance was calculated by comparing the peak areas of a single metabolite to the sum of the peak areas of all given metabolites. Relative abundance = (peak area of a single metabolite/total peak area of metabolites) × 100%.

**Table 2:** Accurate quality detection information of **PF74** and its metabolites in HLMs.

Metabolite	Formula	Exact Mass	Measured Value (*m*/*z*)	Theoretical Value (*m*/*z*)	PPM
M1	C_16_H_18_N_2_O	254.1419	255.1490	255.1492	−0.8
M2	C_26_H_25_N_3_O_5_	459.1794	460.1866	460.1867	−0.2
M3	C_26_H_25_N_3_O_5_	459.1794	460.1866	460.1867	−0.2
M4	C_27_H_27_N_3_O_5_	473.1951	474.2017	474.2024	−1.5
M5	C_27_H_27_N_3_O_5_	473.1951	474.2028	474.2024	0.8
M6	C_26_H_25_N_3_O_5_	459.1794	460.1866	460.1867	−0.2
M7	C_26_H_25_N_3_O_4_	443.1845	444.1917	444.1918	−0.2
M8	C_26_H_25_N_3_O_5_	459.1794	460.1867	460.1867	0.0
M9	C_27_H_27_N_3_O_4_	457.2002	458.2074	458.2075	−0.2
M10	C_26_H_25_N_3_O_5_	459.1794	460.1863	460.1867	−0.9
M11	C_26_H_25_N_3_O_4_	443.1845	444.1917	444.1918	−0.2
M12	C_26_H_25_N_3_O_5_	459.1794	460.1864	460.1867	−0.7
M13	C_26_H_25_N_3_O_4_	443.1845	444.1918	444.1918	0.0
M14	C_26_H_25_N_3_O_4_	443.1845	444.1915	444.1918	−0.7
M15	C_27_H_27_N_3_O_3_	441.2052	442.2134	442.2125	2.0
M16	C_26_H_25_N_3_O_4_	443.1845	444.1919	444.1918	0.2
M17	C_26_H_25_N_3_O_3_	427.1896	428.1969	428.1969	0.0
PF74	C_27_H_27_N_3_O_2_	425.2103	426.2169	426.2176	−1.6

Theoretical value = exact mass + 1.0073 or − 1.0073; PPM = (measured value − theoretical value)/theoretical value × 10^6^.

**Table 3:** List of putative metabolites of **11L** with the primary identification parameters and hypothesized biotransformation. The relatively most intense metabolites are in bold.

Metabolite	[M + H]^+^/[M − H]^−^ *m*/*z*	Retention Time (min)	Relative Abundance (UV Peak Area%)	UV Peak Area (240–320 nm)	Type of Biotransformation	Diagnostic Ions
M1′	628.2078	8.29	5.36	7217	Tri-oxidation (P + 3O)	628.21, 284.07, 138.09
M2′	612.2122	8.49	5.36	7223	Di-oxidation (P + 2O)	612.21, 475.13, 296.07, 268.08, 138.09
M3′	582.2014	8.56	6.81	9171 ^@^	Mono-oxidation and demethylation (P + O– CH_2_)	582.20, 443.14, 296.07, 268.08, 140.07,
M4′	598.1962	8.60	0.77	1043 ^@^	Di-oxidation and demethylation (P+ 2O − CH_2_)	598.20, 475.13, 312.07, 172.01, 122.06
M5′	552.1917	8.79	4.94	6649	*N*- demethylation and *O*- demethylation (P − 2CH_2_)	552.19, 443.14, 296.07, 268.08
**M6′**	**612.2105**	**8.82**	**12.01**	**16,172**	**Di-oxidation (P + 2O)**	**612.21, 475.13, 312.07, 172.01**
M7′	596.2166	9.05	6.62	8923 ^@^	Mono-oxidation (P + O)	596.22, 459.13, 296.07, 268.08, 138.09
M8′	566.2041	9.08	2.25	3028 ^@^	*N*- demethylation (P − CH_2_)	566.20, 124.08
M9′	580.1873 *	9.78	2.59	3493	Mono-oxidation and demethylation (P + O– CH_2_)	580.19, 156.01
M10′	580.1874 *	9.83	2.62	3533	Mono-oxidation and demethylation (P + O– CH_2_)	580.19, 156.01
**M11′**	**582.2020**	**9.98**	**8.77**	**11,814**	**Mono-oxidation and demethylation (P + O − CH_2_)**	**582.20, 172.01, 122.06**
M12′	594.2021	10.04	2.79	3755	Mono-oxidation and dehydrogenation (P + O − 2H)	594.20, 254.06, 172.01
M13′	610.1976 *	10.06	3.27	4410	Di-oxidation (P + 2O)	610.20, 270.06, 172.01
M14′	566.2068	10.29	3.55	4777 ^# @^	Demethylation (P − CH_2_)	566.20, 443.14, 296.07, 268.08, 156.01
**M15′**	**597.2002**	**10.32**	**15.98**	**21,520 ^# @^**	**Mono-oxidation and oxidative deamination** **(P + O − NH + O)**	**597.20, 579.21, 313.05, 285.05**
**M16′**	**596.2158**	**10.36**	**9.20**	**12,391 ^# @^**	**Mono-oxidation (P + O)**	**596.22, 312.07, 284.07**
11L (Parent)	580.2224/578.2077	10.69	7.11	9581	NA	580.22, 443.14, 415.14, 296.07, 268.08, 240.08 (_+_); 578.21, 441.12, 311.08, 294.06, 254.06, 228.04, 156.01, 92.05 (−)

The semiquantitative data of UV (240–320 nm) peak area are shown in the table; P, parent; *, the measured values of M9′, M10′, and M13′ (*m/z*) were obtained using the negative mode data; #, peak area is the value after deducting blank matrix; @, M3′, M4′, M14′–M16′ were separated by cyano columns, M7′ and M8′ were separated by phenyl columns; NA, not applicable. Relative abundance was calculated by comparing the peak areas of a single metabolite to the sum of the peak areas of all given metabolites. Relative abundance = (peak area of a single metabolite/total peak area of metabolites) × 100%.

**Table 4:** Accurate quality detection information of **11L** and its metabolites in HLMs.

Metabolite	Formula	Exact Mass	Measured Value	Theoretical Value	PPM
			(*m*/*z*)	(*m*/*z*)	
M1′	C_29_H_33_N_5_O_9_S	627.1999	628.2078	628.2072	1.0
M2′	C_29_H_33_N_5_O_8_S	611.2050	612.2122	612.2123	−0.2
M3′	C_28_H_31_N_5_O_7_S	581.1944	582.2014	582.2017	−0.5
M4′	C_28_H_31_N_5_O_8_S	597.1893	598.1962	598.1966	−0.7
M5′	C_27_H_29_N_5_O_6_S	551.1839	552.1917	552.1912	0.9
M6′	C_29_H_33_N_5_O_8_S	611.2050	612.2105	612.2123	−2.9
M7′	C_29_H_33_N_5_O_7_S	595.2101	596.2166	596.2174	−1.3
M8′	C_28_H_31_N_5_O_6_S	565.1995	566.2041	566.2068	−4.8
M9′	C_28_H_31_N_5_O_7_S	581.1944	580.1873 *	580.1871	0.3
M10′	C_28_H_31_N_5_O_7_S	581.1944	580.1874 *	580.1871	0.5
M11′	C_28_H_31_N_5_O_7_S	581.1944	582.2020	582.2017	0.5
M12′	C_29_H_31_N_5_O_7_S	593.1944	594.2021	594.2017	0.7
M13′	C_29_H_33_N_5_O_8_S	611.2050	610.1976 *	610.1977	−0.2
M14′	C_28_H_31_N_5_O_6_S	565.1995	566.2068	566.2068	0.0
M15′	C_29_H_32_N_4_O_8_S	596.1941	597.2002	597.2014	−2.0
M16′	C_29_H_33_N_5_O_7_S	595.2101	596.2158	596.2174	−2.7
11L	C_29_H_33_N_5_O_6_S	579.2152	580.2224	580.2225	−0.2

*, The measured values (*m/z*) of M9′, M10′, and M13′ are based on the data of the negative ion mode. Theoretical value = exact mass + 1.0073 or − 1.0073; PPM = (measured value − theoretical value)/theoretical value × 10^6^.

## Data Availability

The data presented in this study are available in the article.

## Supplementary Materials

The following are available online at https://www.mdpi.com/article/10.3390/metabo12080752/s1, Scheme S1: The synthetic route of 11L. Table S1: Metabolic rate of 7-EC in HLMs (%). Figure S1: UPLC–UV chromatograms of 7-EC and its metabolites in HLMs (wavelength: 290–360 nm). Figure S2: Collision-induced dissociation (CID) spectra and proposed fragmentation patterns for metabolites of **PF74**. Figure S3: CID spectra and proposed fragmentation patterns for metabolites of **11L**.
