# Supplementary material for: Metabolite Identification of HIV-1 Capsid Modulators PF74 and 11L in Human Liver Microsomes
Source: Metabolites. 2022 Aug 16;12(8):752. doi: 10.3390/metabo12080752 (PMC9412436; doi:10.3390/metabo12080752)
Supplement: Supplementary file 1 [file metabolites-12-00752-s001.zip › metabolites-1834292-supplementary.pdf]

Supplementary Materials

1. The detailed information of 11L [1].

1.1 The synthetic route of 11L.

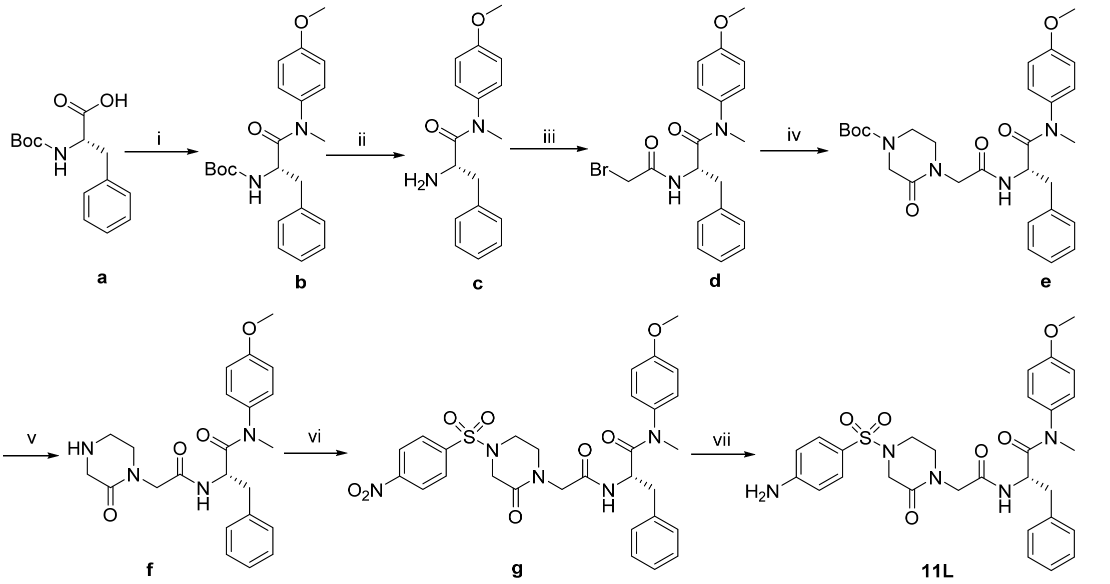

Scheme S1. The synthetic route of **11L**. Reagents and conditions:(i) 4-Methoxy-*N*-methylaniline, HATU, DIEA, 0°C to r.t.; (ii) TFA, DCM, r.t.; (iii) Bromoacetic acid, HATU, DIEA, 0°C to r.t.; (iv) 1-Boc-3-oxopiperazine, Cs_2CO_3, DMF, 45°C; (v) TFA, DCM, r.t.; (vi) TEA, DCM, r.t.; (vii) H_2, 10%Pd•C, DCM, r.t..

11L was prepared according to the general **Scheme S1.** Briefly, the commercially available (*tert*-butoxycarbonyl)-*L*-phenylalanine (**a**) was treated with 4-methoxy-*N*-methylaniline and 2-(7-aza-1H-benzotriazole-1-yl)-1,1,3,3-tetramethyluronium hexafluoro (HATU) in the presence of *N*, *N*-diisopropylethyl-amine (DIEA) and dichloromethane (DCM) to give **b**, followed by the removal of *tert*-butyloxycarbonyl (Boc) protection using trifluoroacetic acid (TFA) to produce the free amine **c**. The acylation of **c** with bromoacetic acid in dichloromethane solution resulted in the key intermediate **d**. Further treatment of **d** with 1-Boc-3-oxopiperazine *via* a nucleophilic substitution (SN_2) reaction to produce intermediate **e**. Removal of the Boc protection of **e** yield the free amine **f**, which was reacted with *p*-nitro benzenesulfonyl chloride by an acylation reaction to give the compound **g**. The amino analogues **11L** was prepared by a hydrogenation reduction of the nitro group of **g**.

1.2 Spectral data of 11L.

(*S*)-2-(2-(4-((4-aminophenyl)sulfonyl)-2-oxopiperazin-1-yl)acetamido)-*N*-(4-methoxyphenyl)-*N*-methyl-3-phenylpropanamide (11L). White solid, yield: 61%. mp: 128-129ºC. ^{1}H NMR (400 MHz, DMSO-d_6) δ 8.37 (d, J = 7.9 Hz, 1H, NH), 7.42 (d, J = 8.6 Hz, 2H, Ph-H), 7.30 – 7.15 (m, 3H, Ph-H), 7.12 (d, J = 7.9 Hz, 2H, Ph-H), 6.98 (d, J = 8.8 Hz, 2H, Ph-H), 6.88 – 6.79 (m, 2H, Ph-H), 6.68 (d, J = 8.7 Hz, 2H, Ph-H), 6.20 (s, 2H, NH_2), 4.44 (td, J = 8.8, 5.2 Hz, 1H, CH), 3.86 (s, 2H, piperazine-CH_2), 3.79 (s, 3H, OCH_3), 3.46 – 3.36 (m, 2H, piperazineCH_2), 3.23 – 3.12 (m, 2H, piperazine-CH_2), 3.10 (s, 3H, NCH_3), 3.07 – 2.95 (m, 2H, piperazine-CH_2), 2.84 (dd, J = 13.5, 4.8 Hz, 1H, PhCH), 2.63 (dd, J = 13.4, 9.5 Hz, 1H, PhCH). ^{13}C NMR (100 MHz, DMSO-d_6) δ 171.38 (C=O), 167.38 (C=O), 163.98 (C=O), 158.99, 154.17, 137.91, 135.90, 130.31, 129.29, 129.14, 128.58, 126.88, 118.49, 115.14, 113.30, 55.88, 51.83, 49.30, 48.38, 46.94, 43.32, 37.77, 37.66. HRMS: m/z 580.2228 $(M+1)^+$, 1181.4192 $(2M+23)^+$. $C_{29}H_{33}N_5O_6S$ [579.2152].

2. Chromatographic and Mass Spectrometric Methods of 7-EC

The UPLC-UV-HRMS analysis was performed on an ACQUITY UPLC system hyphenated to a Thermo Q-Exactive HF tandem mass spectrometer equipped with an electrospray ionization interface operating in positive ion mode.

Chromatographic Method: The chromatography was carried out on an ACQUITY UPLC HSS T3 column (2.1×100 mm, 1.8 μm; Waters Corporation, MA, USA) maintained at 40℃. Automatic injector temperature maintained at 4 ℃. The mobile phase consisting of water containing 0.1 % formic acid (A) and acetonitrile solution containing 0.1 % formic acid (B) was delivered at a flow rate of 300 μL/min. The gradient procedures were optimized as follows: 2% B at 0–1 min, 2%–15% B at 1–8 min, 15%–40% B at 8–14 min, 40%–80% B at 14–16 min, 90% B at 16–18 min and finally 2% B at 18–20 min. The injection volume was 10 μL. UV detection was performed using a photodiode array detector, and wavelength was 190~500 nm.

Mass Spectrometric Method: The electrospray ionization source parameters were set as follows: capillary temperature, 375 ℃; spray voltage, 3.5 kV; sheath gas flow rate,40 L/h; auxiliary gas flow rate, 15 L/h; auxiliary gas heater temperature, 350℃; S-Lens voltage, 55 V. The data were acquired in the m/z range of 80–1200 Da (full mass scan) and 200-2000 Da

(MS/MS scan) in centroid mode. The collision energy was 10/15/25 V. The mass resolution for full mass scan and MS/MS scan was selected to be above 120000 and 30000, respectively. Collision-induced dissociation (CID) was used to obtain the MS/MS fragment of 7-EC and its metabolites. All the data acquisition was obtained by Xcalibur software (version 4.2; Thermo Fisher Scientific).

Table S1 Metabolism rate of 7-EC in HLMs (%)

**Species**	**UV peak area in T0 (wavelength：290-360 nm)**	**UV peak area in T60 (wavelength：290-360 nm)**	**Metabolism rate (%)**
**Human**	245954	0	100

Note: Metabolism rate (%) = (1- UV peak area in T60/ UV peak area in T0)×100%

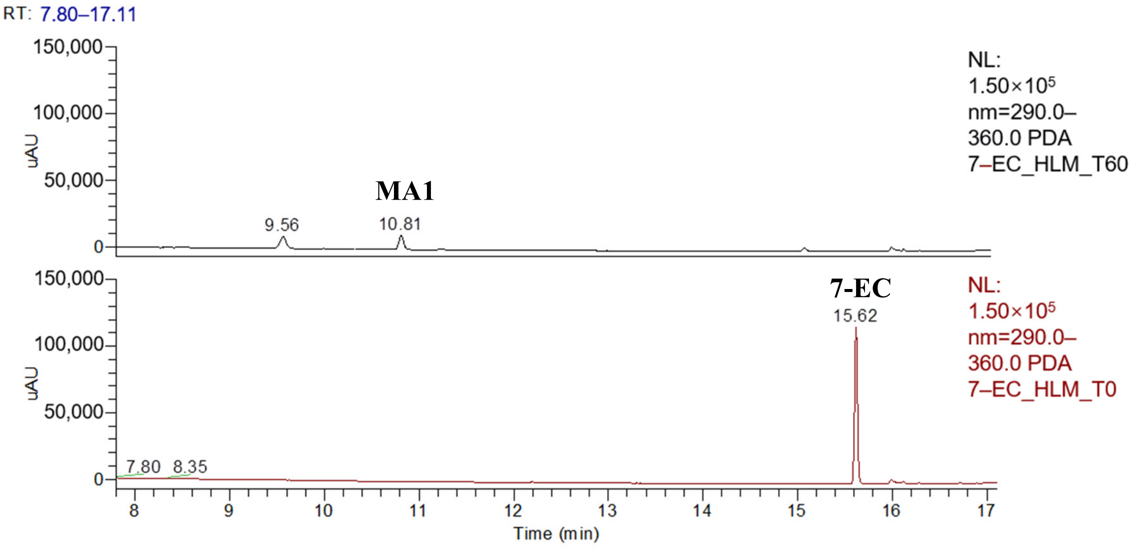

Figure S1 UPLC-UV chromatograms of 7-EC and its metabolites in HLMs (wavelength: 290 – 360 nm)

Note: MA1 was a deethyl metabolite.

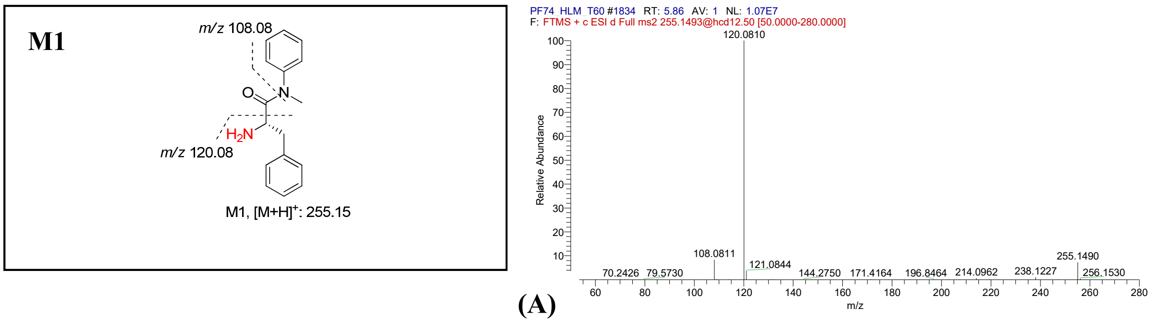
M1
m/z 108.08
m/z 120.08
M1, [M+H]+: 255.15
PF74 HLM T60 #1834 RT: 5.86 AV: 1 NL: 1.07E7
F: FTMS + c ESI d Full ms2 255.1493@hcd12.50 [50.0000-280.0000]
Relative Abundance
120.0810
108.0811
121.0844
70.2426
79.5730
144.2750
171.4164
196.8464
214.0962
238.1227
255.1490
256.1530
m/z

(A)

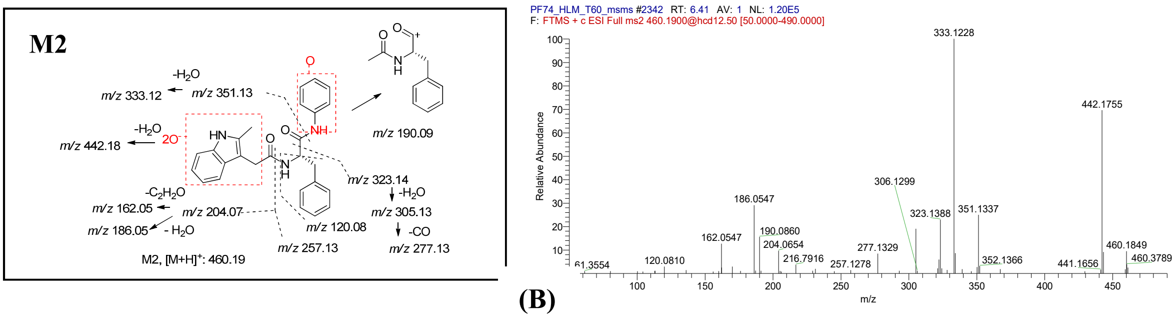
M2
m/z 333.12
m/z 351.13
m/z 442.18
m/z 162.05
m/z 186.05
m/z 204.07
m/z 120.08
m/z 257.13
m/z 323.14
m/z 305.13
m/z 277.13
m/z 190.09
M2, [M+H]+: 460.19
PF74_HLM_T60_msms #2342 RT: 6.41 AV: 1 NL: 1.20E5
F: FTMS + c ESI Full ms2 460.1900@hcd12.50 [50.0000-490.0000]
Relative Abundance
333.1228
442.1755
306.1299
186.0547
323.1388
351.1337
162.0547
190.0860
204.0654
216.7916
257.1278
277.1329
352.1366
441.1656
460.1849
460.3789
120.0810
m/z

(B)

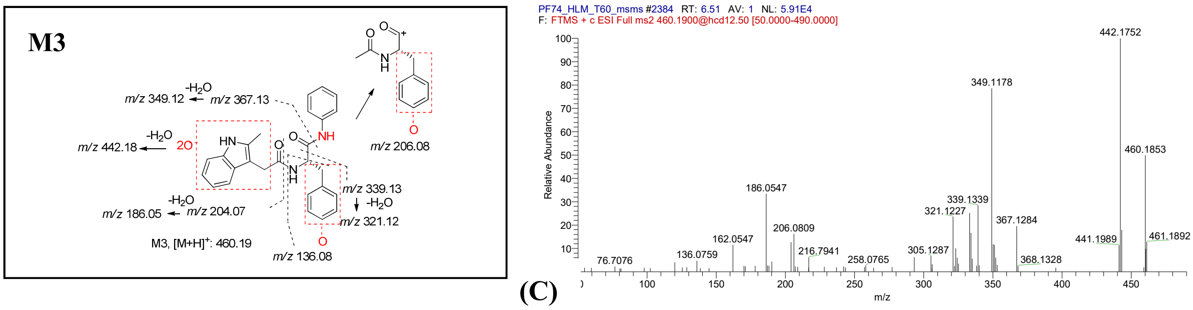
M3
m/z 349.12
m/z 367.13
m/z 442.18
m/z 186.05
m/z 204.07
m/z 339.13
m/z 321.12
m/z 136.08
m/z 206.08
M3, [M+H]+: 460.19
PF74_HLM_T60_msms #2384 RT: 6.51 AV: 1 NL: 5.91E4
F: FTMS + c ESI Full ms2 460.1900@hcd12.50 [50.0000-490.0000]
Relative Abundance
442.1752
349.1178
460.1853
186.0547
339.1339
321.1227
367.1284
206.0809
162.0547
216.7941
136.0759
76.7076
258.0765
305.1287
368.1328
441.1989
461.1892
m/z

(C)

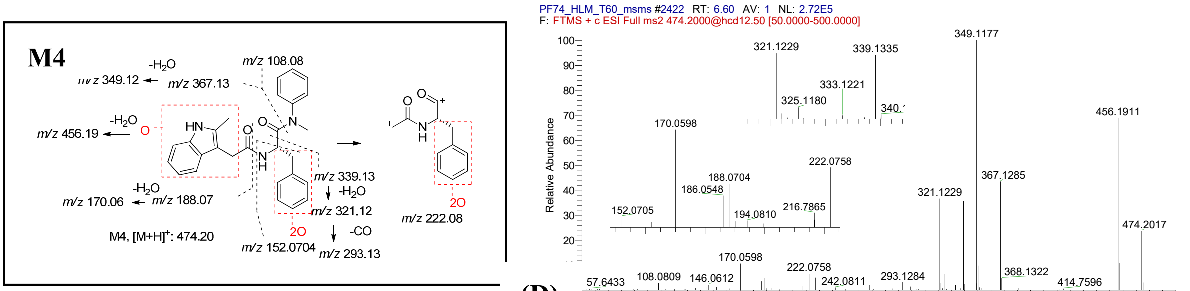
M4
m/z 108.08
m/z 349.12
m/z 367.13
m/z 456.19
m/z 170.06
m/z 188.07
m/z 339.13
m/z 321.12
m/z 293.13
m/z 152.0704
m/z 222.08
M4, [M+H]+: 474.20
PF74_HLM_T60_msms #2422 RT: 6.60 AV: 1 NL: 2.72E5
F: FTMS + c ESI Full ms2 474.2000@hcd12.50 [50.0000-500.0000]
Relative Abundance
349.1177
321.1229
339.1335
333.1221
325.1180
170.0598
456.1911
222.0758
188.0704
186.0548
194.0810
216.7865
152.0705
367.1285
474.2017
368.1322
414.7596
57.6433
108.0809
146.0612
242.0811
293.1284
m/z

(D)

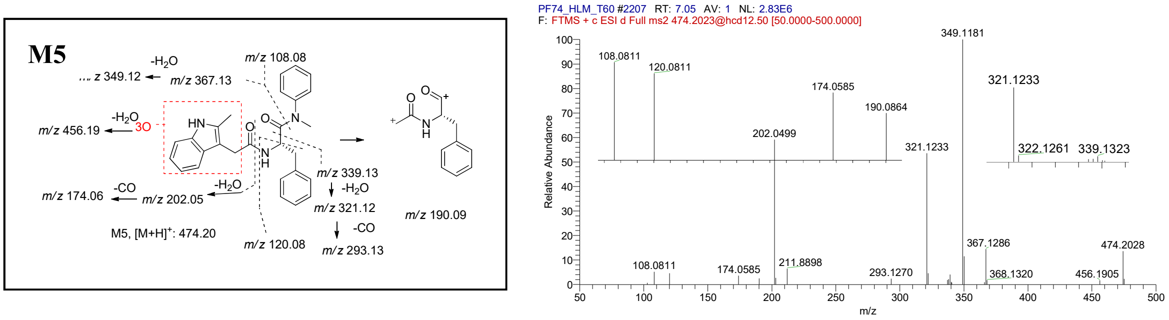
M5
m/z 108.08
m/z 367.13
m/z 456.19
m/z 174.06
m/z 202.05
m/z 339.13
m/z 321.12
m/z 293.13
m/z 120.08
m/z 190.09
M5, [M+H]+: 474.20
PF74_HLM_T60 #2207 RT: 7.05 AV: 1 NL: 2.83E6
F: FTMS + c ESI d Full ms2 474.2023@hcd12.50 [50.0000-500.0000]
Relative Abundance
349.1181
108.0811
120.0811
174.0585
190.0864
202.0499
321.1233
322.1261
339.1323
211.8898
293.1270
367.1286
368.1320
456.1905
474.2028
m/z

(E)

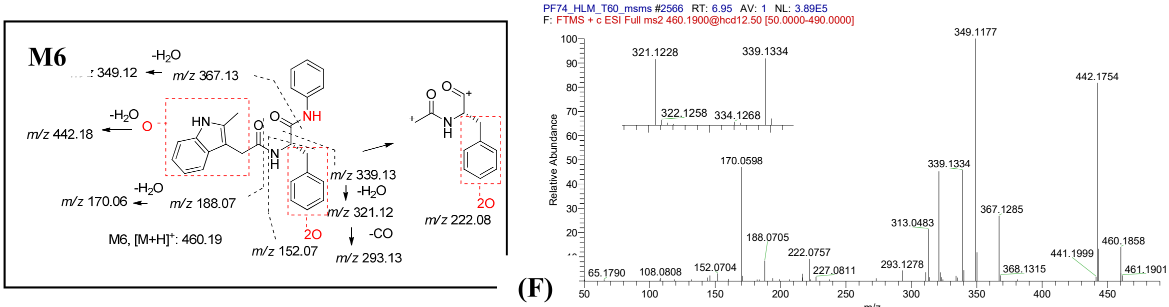
M6
-H2O
349.12
m/z 367.13
-H2O
m/z 442.18
O
HN
O
NH
-H2O
m/z 170.06
m/z 188.07
N H
m/z 339.13
-H2O
m/z 321.12
-CO
m/z 293.13
2O
M6, [M+H]+: 460.19
m/z 152.07
2O
m/z 222.08
PF74_HLM_T60_msms #2566 RT: 6.95 AV: 1 NL: 3.89E5
F: FTMS + c ESI Full ms2 460.1900@hcd12.50 [50.0000-490.0000]
Relative Abundance
m/z
349.1177
321.1228
339.1334
322.1258
334.1268
442.1754
170.0598
339.1334
367.1285
313.0483
188.0705
460.1858
222.0757
441.1999
293.1278
65.1790
108.0808
152.0704
227.0811
368.1315
461.1901
(F)

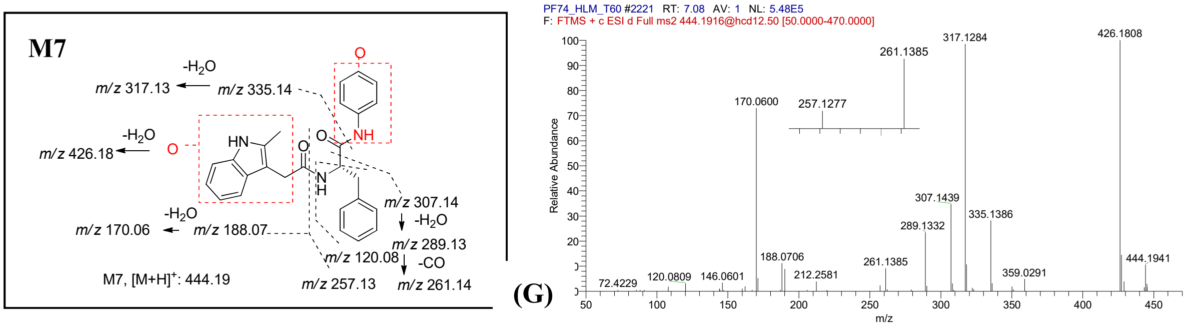
M7
-H2O
m/z 317.13
m/z 335.14
O
-H2O
m/z 426.18
O
HN
O
NH
N H
-H2O
m/z 170.06
m/z 188.07
m/z 307.14
-H2O
m/z 289.13
m/z 120.08
-CO
M7, [M+H]+: 444.19
m/z 257.13
m/z 261.14
PF74_HLM_T60 #2221 RT: 7.08 AV: 1 NL: 5.48E5
F: FTMS + c ESI d Full ms2 444.1916@hcd12.50 [50.0000-470.0000]
Relative Abundance
m/z
317.1284
426.1808
261.1385
170.0600
257.1277
307.1439
335.1386
289.1332
188.0706
261.1385
444.1941
72.4229
120.0809
146.0601
212.2581
359.0291
(G)

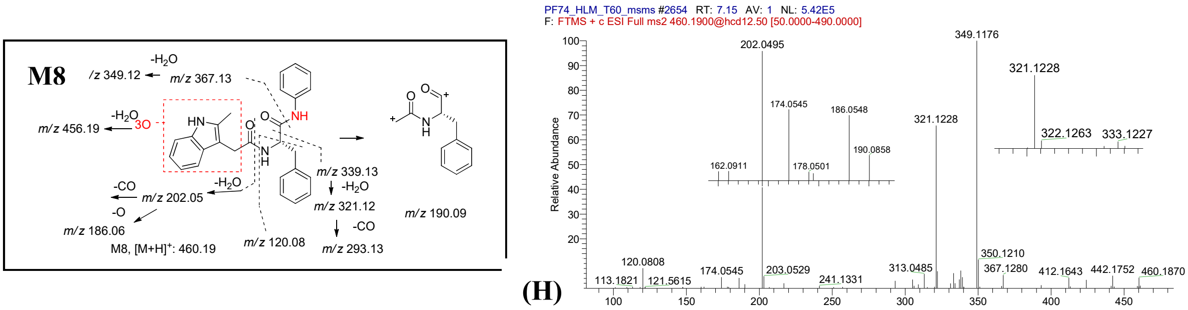
M8
-H2O
/z 349.12
m/z 367.13
-H2O
m/z 456.19
3O
HN
O
NH
N H
m/z 339.13
-H2O
m/z 321.12
-CO
m/z 293.13
-CO
m/z 202.05
-H2O
-O
m/z 186.06
M8, [M+H]+: 460.19
m/z 120.08
m/z 190.09
PF74_HLM_T60_msms #2654 RT: 7.15 AV: 1 NL: 5.42E5
F: FTMS + c ESI Full ms2 460.1900@hcd12.50 [50.0000-490.0000]
Relative Abundance
m/z
349.1176
202.0495
321.1228
174.0545
186.0548
322.1263
333.1227
321.1228
190.0858
162.0911
178.0501
120.0808
350.1210
113.1821
121.5615
174.0545
203.0529
241.1331
313.0485
367.1280
412.1643
442.1752
460.1870
(H)

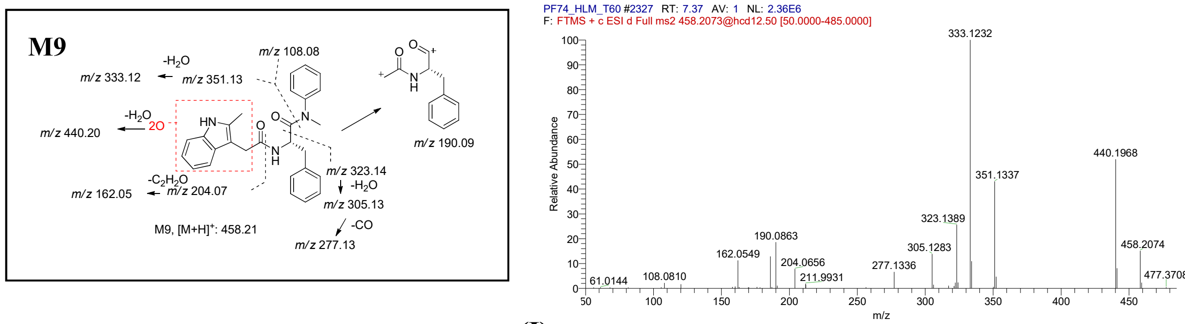
M9
m/z 108.08
-H2O
m/z 333.12
m/z 351.13
-H2O
m/z 440.20
2O
HN
O
N
N H
-C2H2O
m/z 162.05
m/z 204.07
m/z 323.14
-H2O
m/z 305.13
-CO
m/z 277.13
M9, [M+H]+: 458.21
m/z 190.09
PF74_HLM_T60 #2327 RT: 7.37 AV: 1 NL: 2.36E6
F: FTMS + c ESI d Full ms2 458.2073@hcd12.50 [50.0000-485.0000]
Relative Abundance
m/z
333.1232
440.1968
351.1337
323.1389
190.0863
458.2074
162.0549
305.1283
204.0656
277.1336
477.3708
61.0144
108.0810
211.9931
(I)

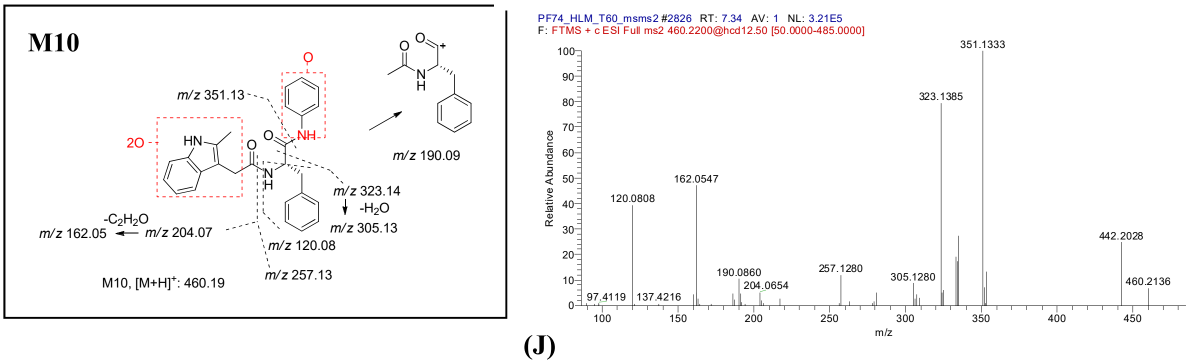
M10
O
m/z 351.13
2O
HN
O
NH
N H
m/z 190.09
m/z 323.14
-H2O
m/z 305.13
-C2H2O
m/z 162.05
m/z 204.07
m/z 120.08
m/z 257.13
M10, [M+H]+: 460.19
PF74_HLM_T60_msms2 #2826 RT: 7.34 AV: 1 NL: 3.21E5
F: FTMS + c ESI Full ms2 460.2200@hcd12.50 [50.0000-485.0000]
Relative Abundance
m/z
351.1333
323.1385
162.0547
120.0808
442.2028
190.0860
257.1280
305.1280
460.2136
97.4119
137.4216
204.0654
(J)

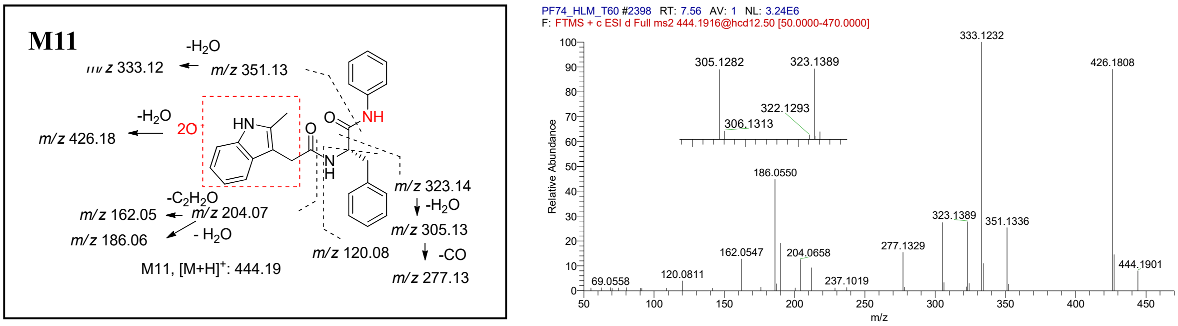
M11
-H_2O
m/z 333.12
m/z 351.13
-H_2O
2O
m/z 426.18
HN
O
O
NH
N
H
-C_2H_2O
m/z 162.05
m/z 204.07
m/z 186.06
- H_2O
m/z 120.08
m/z 323.14
-H_2O
m/z 305.13
-CO
m/z 277.13
M11, $[M+H]^+$: 444.19
PF74_HLM_T60 #2398 RT: 7.56 AV: 1 NL: 3.24E6
F: FTMS + c ESI d Full ms2 444.1916@hcd12.50 [50.0000-470.0000]
Relative Abundance
m/z
333.1232
426.1808
305.1282
323.1389
322.1293
306.1313
186.0550
323.1389
351.1336
277.1329
162.0547
204.0658
444.1901
120.0811
69.0558
237.1019

(K)

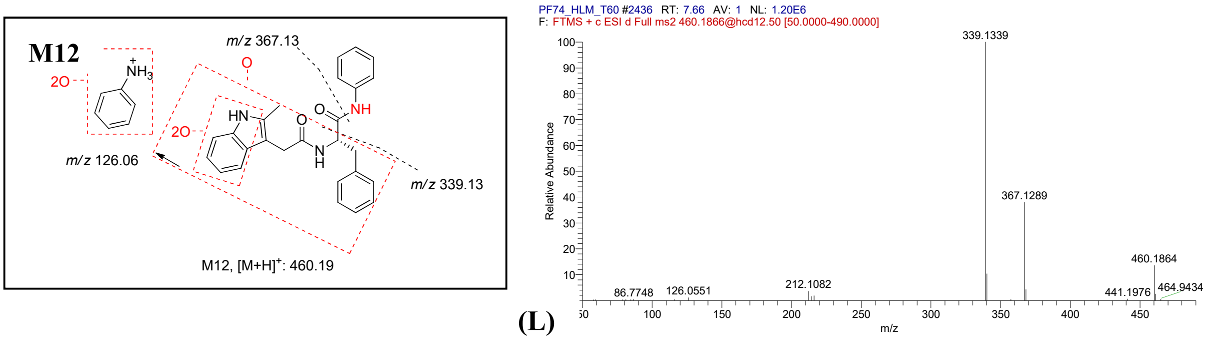
M12
NH_3^+
2O
m/z 126.06
m/z 367.13
O
2O
HN
O
O
NH
N
H
m/z 339.13
M12, $[M+H]^+$: 460.19
PF74_HLM_T60 #2436 RT: 7.66 AV: 1 NL: 1.20E6
F: FTMS + c ESI d Full ms2 460.1866@hcd12.50 [50.0000-490.0000]
Relative Abundance
m/z
339.1339
367.1289
460.1864
441.1976
464.9434
212.1082
86.7748
126.0551

(L)

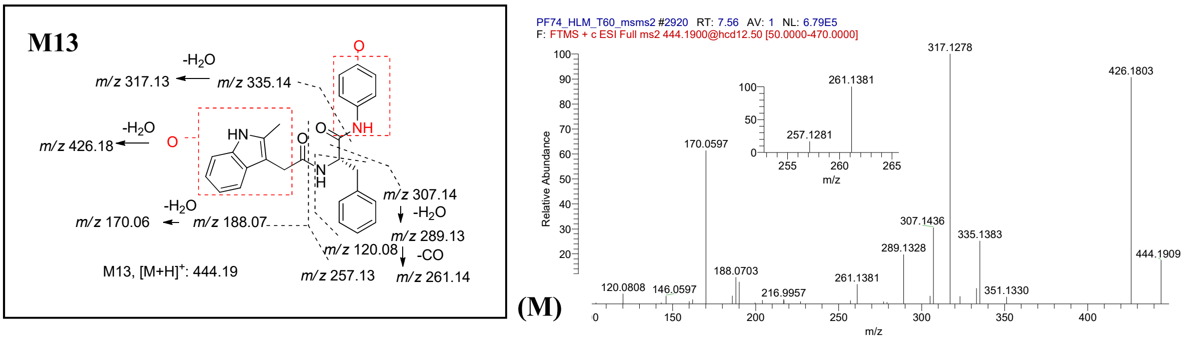
M13
-H_2O
m/z 317.13
m/z 335.14
O
NH
-H_2O
m/z 426.18
O
HN
O
O
N
H
-H_2O
m/z 170.06
m/z 188.07
m/z 307.14
-H_2O
m/z 289.13
m/z 120.08
-CO
m/z 257.13
m/z 261.14
M13, $[M+H]^+$: 444.19
PF74_HLM_T60_msms2 #2920 RT: 7.56 AV: 1 NL: 6.79E5
F: FTMS + c ESI Full ms2 444.1900@hcd12.50 [50.0000-470.0000]
Relative Abundance
m/z
317.1278
426.1803
261.1381
257.1281
170.0597
307.1436
289.1328
335.1383
444.1909
188.0703
261.1381
120.0808
146.0597
216.9957
351.1330

(M)

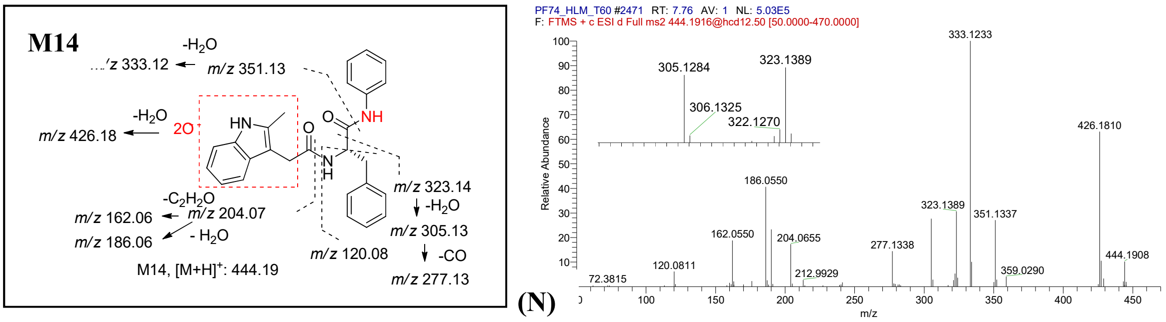
M14
-H_2O
m/z 333.12
m/z 351.13
-H_2O
m/z 426.18
2O
HN
O
O
NH
N
H
-C_2H_2O
m/z 162.06
m/z 204.07
m/z 186.06
- H_2O
m/z 120.08
m/z 323.14
-H_2O
m/z 305.13
-CO
m/z 277.13
M14, $[M+H]^+$: 444.19
PF74_HLM_T60 #2471 RT: 7.76 AV: 1 NL: 5.03E5
F: FTMS + c ESI d Full ms2 444.1916@hcd12.50 [50.0000-470.0000]
Relative Abundance
m/z
333.1233
305.1284
323.1389
306.1325
322.1270
426.1810
186.0550
323.1389
351.1337
162.0550
204.0655
277.1338
444.1908
120.0811
72.3815
212.9929
359.0290

(N)

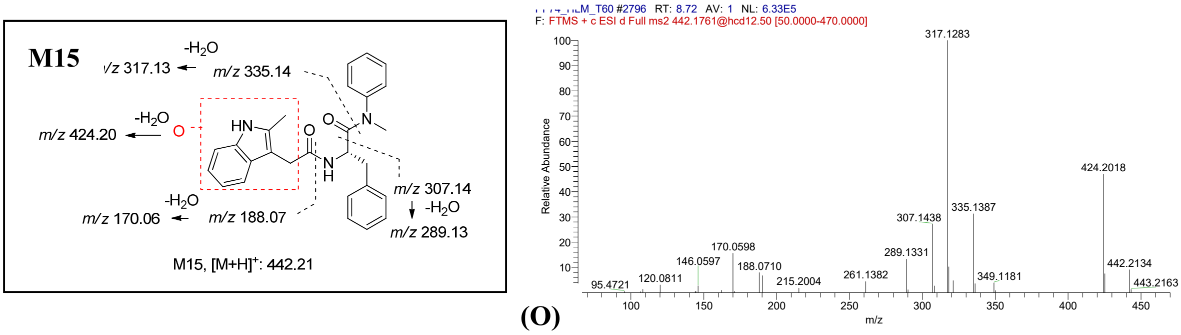
M15
-H_2O
m/z 317.13
m/z 335.14
-H_2O
m/z 424.20
O
HN
O
O
N
N
H
-H_2O
m/z 170.06
m/z 188.07
m/z 307.14
-H_2O
m/z 289.13
M15, $[M+H]^+$: 442.21
_HLM_T60 #2796 RT: 8.72 AV: 1 NL: 6.33E5
F: FTMS + c ESI d Full ms2 442.1761@hcd12.50 [50.0000-470.0000]
Relative Abundance
m/z
317.1283
424.2018
307.1438
335.1387
170.0598
289.1331
146.0597
188.0710
442.2134
443.2163
120.0811
95.4721
215.2004
261.1382
349.1181

(O)

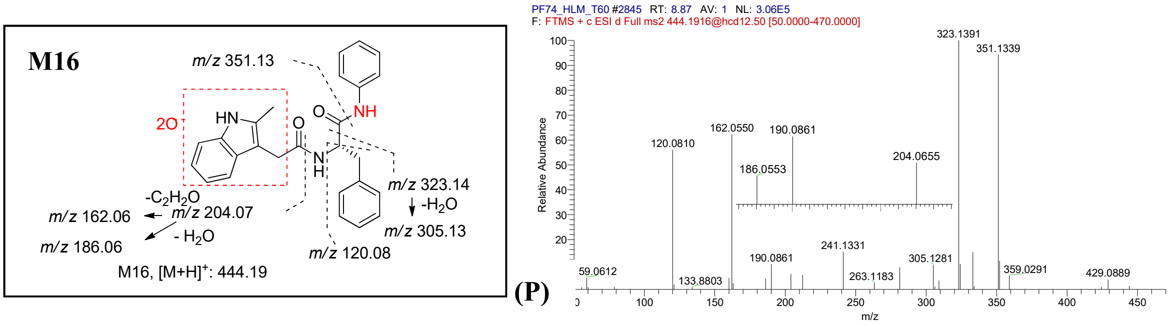

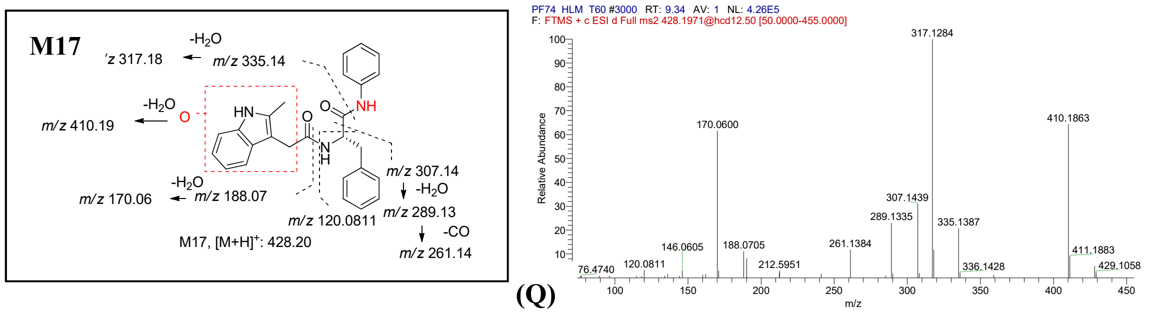

Figure S2. Collision induced dissociation (CID) spectra and proposed fragmentation patterns for metabolites of **PF74**.

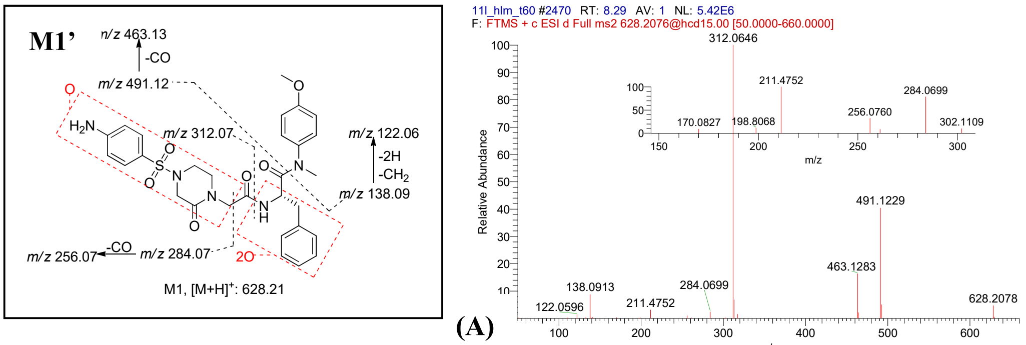

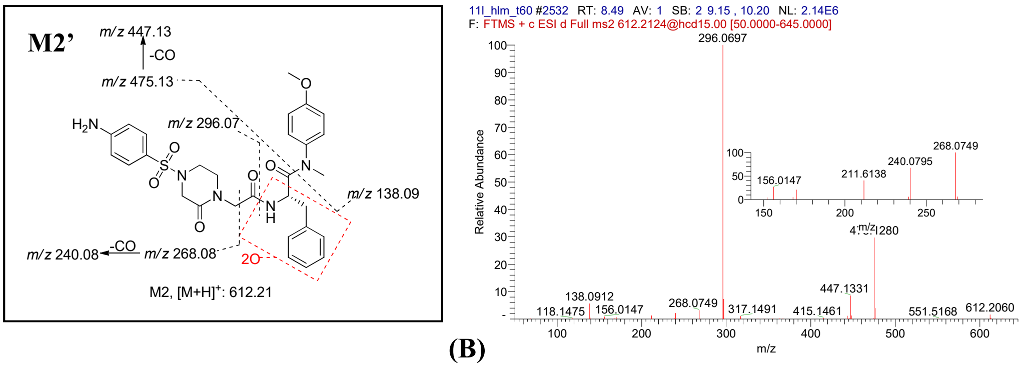

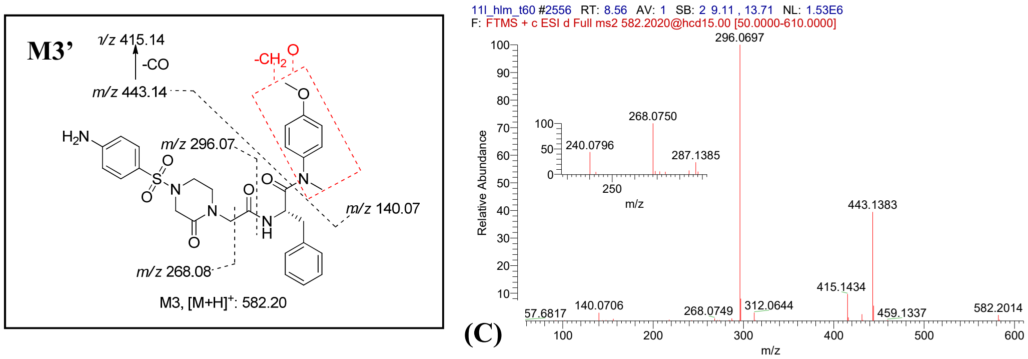
M3'
m/z 415.14
-CO
m/z 443.14
-CH2 O
H2N
m/z 296.07
m/z 140.07
m/z 268.08
M3, [M+H]+: 582.20
11l_hlm_t60 #2556 RT: 8.56 AV: 1 SB: 2 9.11 , 13.71 NL: 1.53E6
F: FTMS + c ESI d Full ms2 582.2020@hcd15.00 [50.0000-610.0000]
Relative Abundance
m/z
296.0697
268.0750
240.0796
287.1385
443.1383
415.1434
57.6817
140.0706
268.0749
312.0644
459.1337
582.2014

(C)

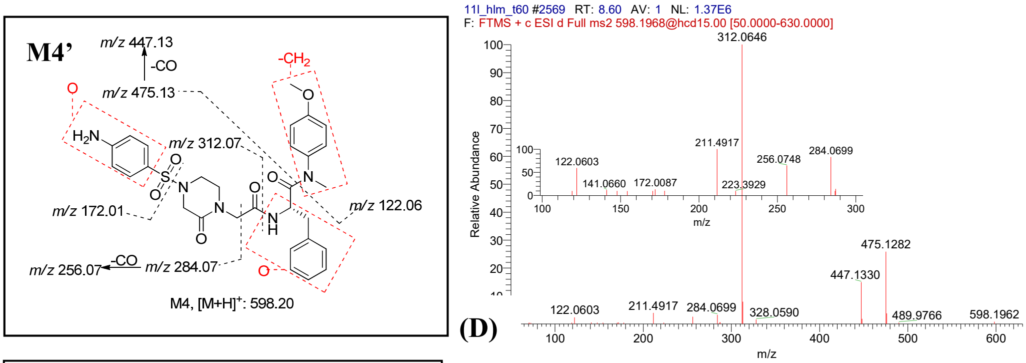
M4'
m/z 447.13
-CO
m/z 475.13
-CH2
O
H2N
m/z 312.07
m/z 172.01
m/z 122.06
m/z 256.07
-CO
m/z 284.07
O
M4, [M+H]+: 598.20
11l_hlm_t60 #2569 RT: 8.60 AV: 1 NL: 1.37E6
F: FTMS + c ESI d Full ms2 598.1968@hcd15.00 [50.0000-630.0000]
Relative Abundance
m/z
312.0646
122.0603
141.0660
172.0087
211.4917
223.3929
256.0748
284.0699
475.1282
447.1330
211.4917
284.0699
328.0590
489.9766
598.1962

(D)

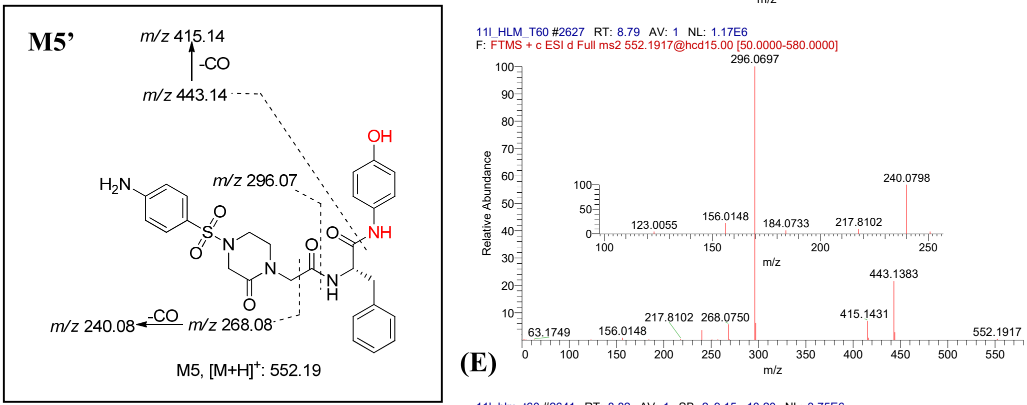
M5'
m/z 415.14
-CO
m/z 443.14
OH
H2N
m/z 296.07
NH
m/z 240.08
-CO
m/z 268.08
M5, [M+H]+: 552.19
11l_HLM_T60 #2627 RT: 8.79 AV: 1 NL: 1.17E6
F: FTMS + c ESI d Full ms2 552.1917@hcd15.00 [50.0000-580.0000]
Relative Abundance
m/z
296.0697
123.0055
156.0148
184.0733
217.8102
240.0798
443.1383
415.1431
63.1749
156.0148
217.8102
268.0750
552.1917

(E)

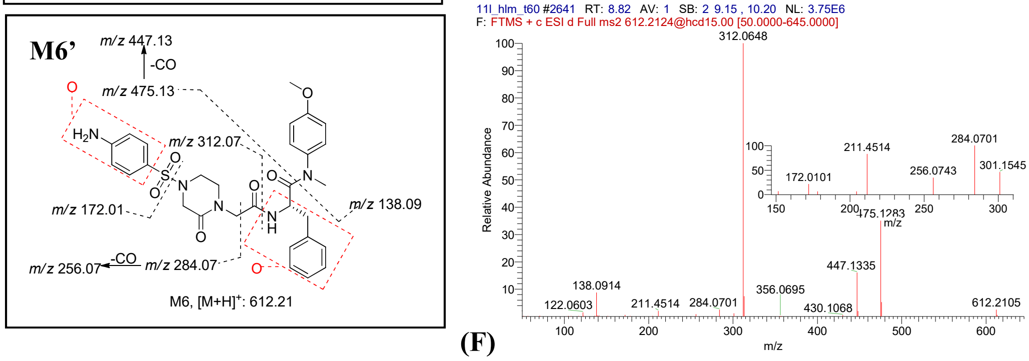
M6'
m/z 447.13
-CO
m/z 475.13
O
H2N
m/z 312.07
m/z 172.01
m/z 138.09
m/z 256.07
-CO
m/z 284.07
O
M6, [M+H]+: 612.21
11l_hlm_t60 #2641 RT: 8.82 AV: 1 SB: 2 9.15 , 10.20 NL: 3.75E6
F: FTMS + c ESI d Full ms2 612.2124@hcd15.00 [50.0000-645.0000]
Relative Abundance
m/z
312.0648
172.0101
211.4514
256.0743
284.0701
301.1545
175.1283
447.1335
138.0914
122.0603
211.4514
284.0701
356.0695
430.1068
612.2105

(F)

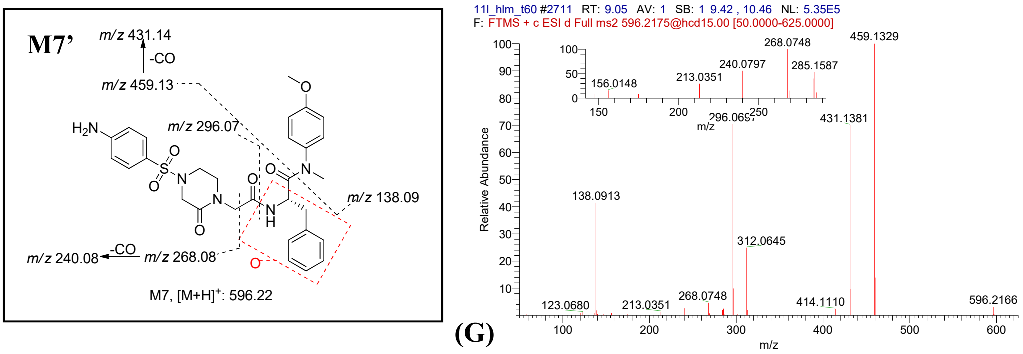
M7'
m/z 431.14
-CO
m/z 459.13
H2N
m/z 296.07
m/z 138.09
m/z 240.08
-CO
m/z 268.08
O
M7, [M+H]+: 596.22
11l_hlm_t60 #2711 RT: 9.05 AV: 1 SB: 1 9.42 , 10.46 NL: 5.35E5
F: FTMS + c ESI d Full ms2 596.2175@hcd15.00 [50.0000-625.0000]
Relative Abundance
m/z
156.0148
213.0351
240.0797
268.0748
285.1587
459.1329
296.0697
431.1381
138.0913
312.0645
123.0680
213.0351
268.0748
414.1110
596.2166

(G)

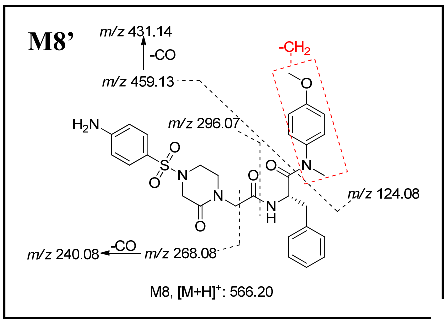
M8’
m/z 431.14
-CO
m/z 459.13
-CH2
m/z 296.07
m/z 124.08
m/z 240.08
-CO
m/z 268.08
M8, [M+H]+: 566.20

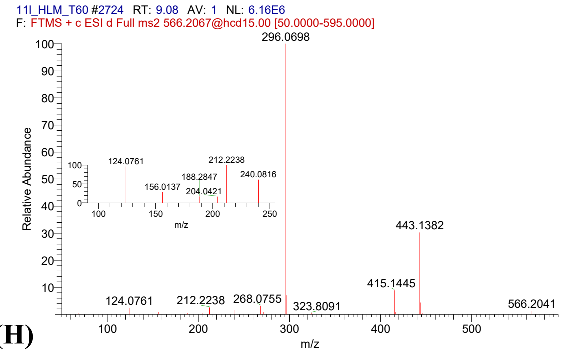
11l_HLM_T60 #2724 RT: 9.08 AV: 1 NL: 6.16E6
F: FTMS + c ESI d Full ms2 566.2067@hcd15.00 [50.0000-595.0000]
296.0698
124.0761
156.0137
188.2847
204.0421
212.2238
240.0816
443.1382
415.1445
124.0761
212.2238
268.0755
323.8091
566.2041
Relative Abundance
m/z

(H)

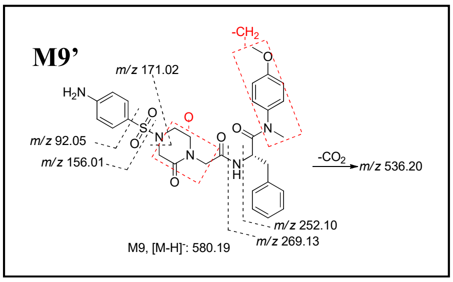
M9’
-CH2
m/z 171.02
m/z 92.05
m/z 156.01
-CO2
m/z 536.20
m/z 252.10
m/z 269.13
M9, [M-H]-: 580.19

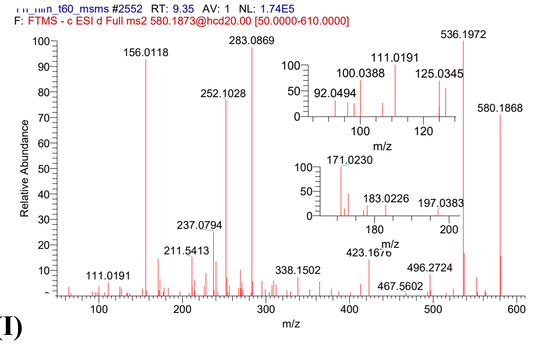
F: FTMS - c ESI d Full ms2 580.1873@hcd20.00 [50.0000-610.0000]
156.0118
283.0869
536.1972
252.1028
580.1868
111.0191
100.0388
125.0345
92.0494
171.0230
183.0226
197.0383
237.0794
211.5413
338.1502
496.2724
467.5602
111.0191
Relative Abundance
m/z

(I)

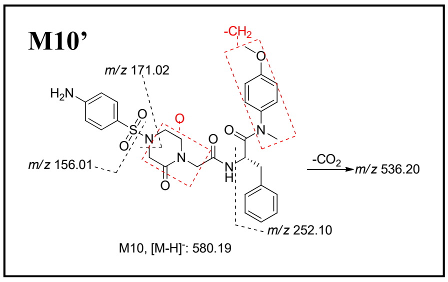
M10’
-CH2
m/z 171.02
m/z 156.01
-CO2
m/z 536.20
m/z 252.10
M10, [M-H]-: 580.19

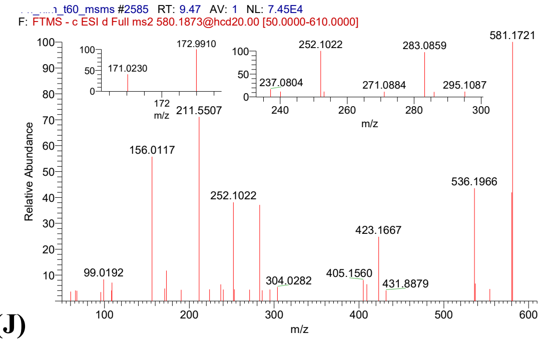
F: FTMS - c ESI d Full ms2 580.1873@hcd20.00 [50.0000-610.0000]
172.9910
171.0230
252.1022
283.0859
237.0804
271.0884
295.1087
581.1721
211.5507
156.0117
536.1966
252.1022
423.1667
99.0192
304.0282
405.1560
431.8879
Relative Abundance
m/z

(J)

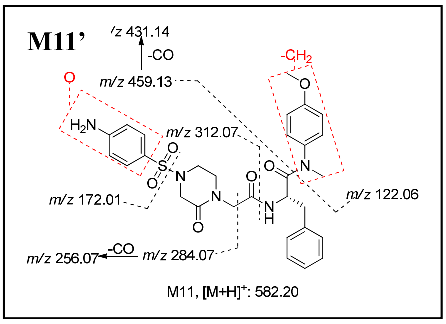
M11’
m/z 431.14
-CO
m/z 459.13
-CH2
m/z 312.07
m/z 172.01
m/z 122.06
m/z 256.07
-CO
m/z 284.07
M11, [M+H]+: 582.20

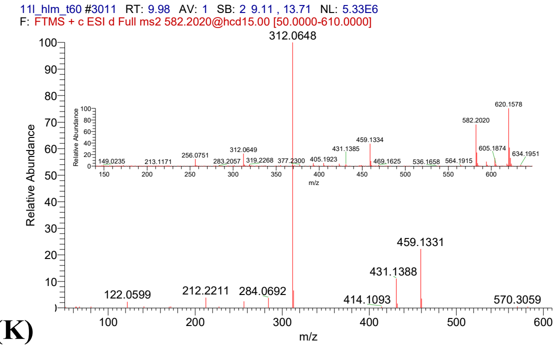
11l_hlm_t60 #3011 RT: 9.98 AV: 1 SB: 2 9.11 , 13.71 NL: 5.33E6
F: FTMS + c ESI d Full ms2 582.2020@hcd15.00 [50.0000-610.0000]
312.0648
459.1331
431.1388
122.0599
212.2211
284.0692
414.1093
570.3059
Relative Abundance
m/z

(K)

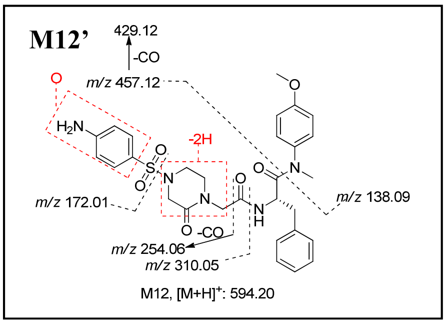
M12’
429.12
-CO
m/z 457.12
-2H
m/z 172.01
m/z 138.09
m/z 254.06
-CO
m/z 310.05
M12, [M+H]+: 594.20

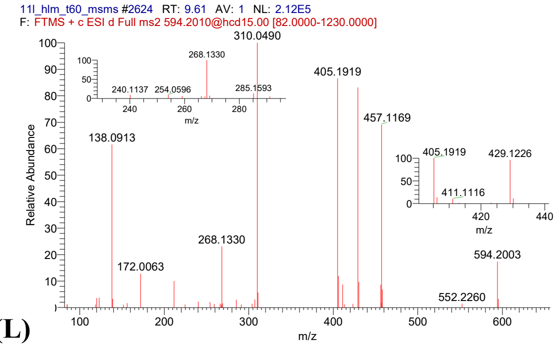
11l_hlm_t60_msms #2624 RT: 9.61 AV: 1 NL: 2.12E5
F: FTMS + c ESI d Full ms2 594.2010@hcd15.00 [82.0000-1230.0000]
310.0490
268.1330
240.1137
254.0596
285.1593
405.1919
457.1169
138.0913
405.1919
429.1226
411.1116
268.1330
172.0063
594.2003
552.2260
Relative Abundance
m/z

(L)

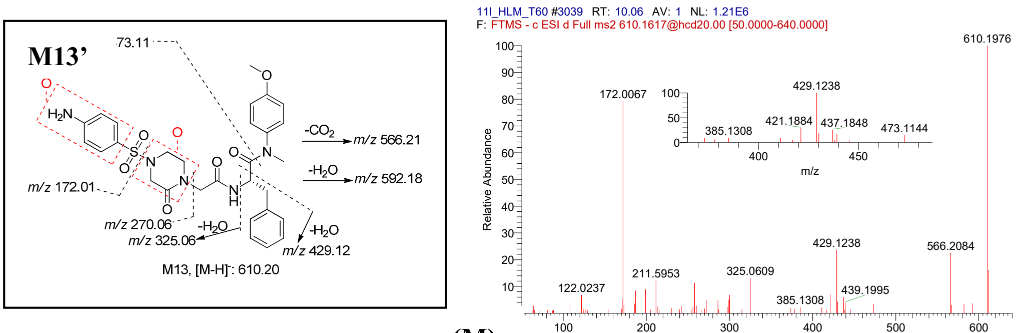

(M)

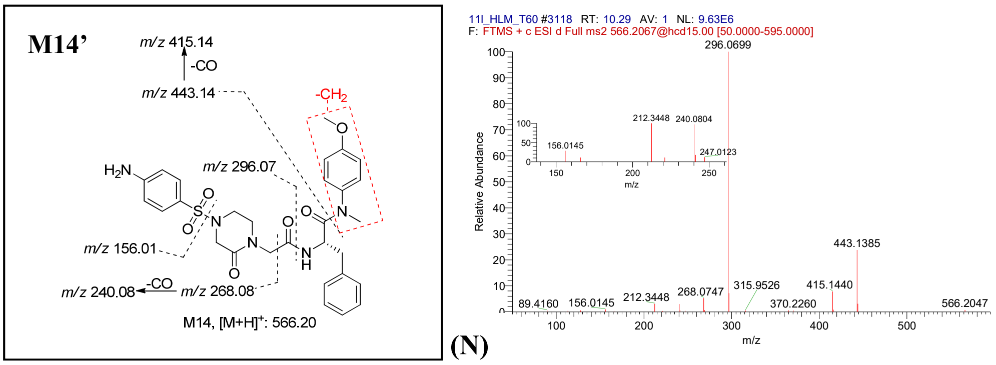

(N)

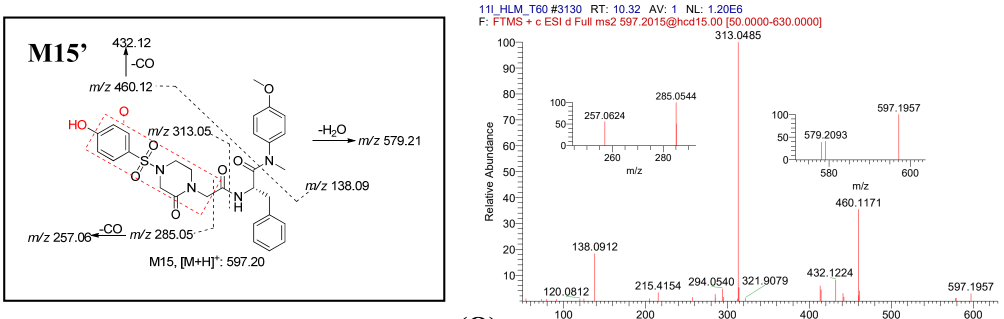

(O)

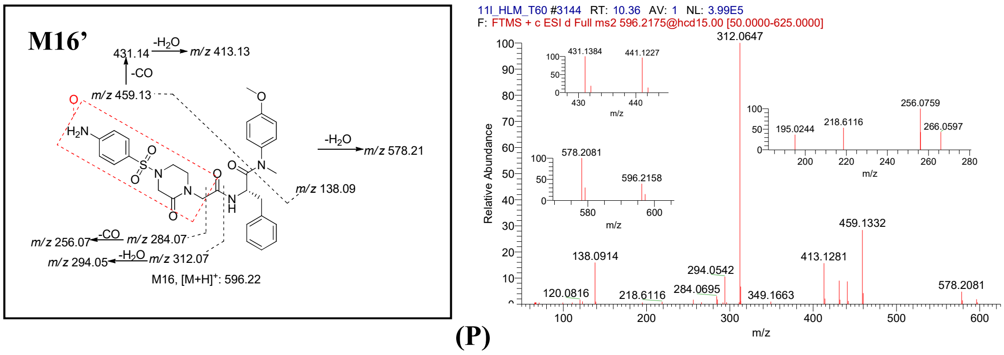

(P)

Figure S3. CID spectra and proposed fragmentation patterns for metabolites of **11L**.

Reference:

[1] Sun L, Dick A, Meuser ME, Huang T, Zalloum WA, Chen CH, Cherukupalli S, Xu S, Ding X, Gao P, Kang D, De Clercq E, Pannecouque C, Cocklin S, Lee KH, Liu X, Zhan P. Design, Synthesis, and Mechanism Study of Benzenesulfonamide-Containing Phenylalanine Derivatives as Novel HIV-1 Capsid Inhibitors with Improved Antiviral Activities. *J. Med. Chem.* **2020**, *63*, 4790-4810
